# A novel extended inverse Weibull distribution: Statistical analysis and application

**DOI:** 10.1371/journal.pone.0335555

**Published:** 2025-10-28

**Authors:** Qin Gong, Ziwen Zhang, Lihua Zeng, Haiping Ren

**Affiliations:** 1 College of Science, Jiangxi University of Science and Technology, Ganzhou, China; 2 Teaching Department of Basic Subjects, Jiangxi University of Science and Technology, Nanchang, China; Shanghai Jiao Tong University, CHINA

## Abstract

This paper proposes a new type of exponential-type Weibull distribution based on the inverse Weibull distribution --- the transformed inverse Weibull distribution. This distribution constructs a more flexible parameter structure through mathematical transformation and has a better fitting effect on actual data. We deeply analyzed the key statistical properties of this distribution, including the probability density function, survival function, quantile function, as well as Shannon entropy, Rényi entropy, Tsallis entropy, and Mathai-Haubold entropy, etc. In terms of parameter estimation, various parameter estimation methods such as maximum likelihood estimation and Bayesian estimation were adopted to estimate the parameters of the transformed inverse Weibull distribution, and the performance of various parameter estimation methods was evaluated through Monte Carlo simulation. Finally, two sets of real data were applied to verify the applicability and effectiveness of the model in practical applications. The results show that the transformed inverse Weibull distribution exhibits a superior fitting performance in the goodness-of-fit test compared to the Weibull distribution, weighted exponential distribution, exponential Pareto distribution, flexible Weibull distribution, generalized exponential distribution, and generalized inverse exponential distribution.

## 1. Introduction

Probability distribution models hold a central position in the fields of statistics and probability theory. They not only provide a theoretical foundation for data modeling, prediction, parameter estimation, and statistical inference but also play a crucial role in describing random processes, constructing complex statistical models, solving optimization problems, and applications in machine learning and artificial intelligence. Although the research on probability distribution models in the existing literature has reached a relatively mature stage, offering robust theoretical support for addressing problems across various domains, traditional probability distribution models do not always achieve optimal fitting in the process of actual data fitting. In light of this, researchers continue to explore more flexible distribution models by extending and transforming classical models, aiming to enhance their fitting performance to meet the evolving needs of data analysis.

In recent years, some progress has been made in converting classical progressive Weibull distribution (WD) models to improve their flexibility. For instance, Alshanbari et al. [1] proposed a new flexible Weibull extension distribution based on the characteristics of extreme data, which can predict and model extreme observations more effectively. In cases where data sets exhibit mixed state faults, traditional probabilistic models are no longer suitable. Therefore, Khan et al. [2] introduced a beta power WD model that is sufficiently flexible to handle multiple failure modes. Al-Marzouki et al. [3] artificially enhanced the accuracy of modeling and prediction by extending the flexible WD (FWD) to develop the modified FWD, and demonstrated through real data verification that its fitting effectiveness surpassed that of the original FWD. Liu et al. [4] proposed a new power FWD by amalgamating the FWD with a novel power transformation method. Simulation studies have shown that this new distribution boasts higher flexibility and superior fitting capabilities compared to the flexible WD. Shi et al. [5] introduced the exponential flexible WD, which integrates the flexible Weibull expansion with the exponential T-X strategy, and it was shown to offer greater flexibility and improved fitting performance over the traditional WD. Gemeay et al. [6] extended the II Laplace semi-logarithmic distribution through power transformation techniques and proposed the power-type II Laplace semi-logarithmic distribution. This new distribution enhances the flexibility and applicability of the original distribution, enabling it to adapt to more complex real-world scenarios. Chaisee et al. [7] proposed a new Gamma-Exponential Weibull Poisson distribution, an extension of the exponential-Weibull Poisson distribution family. It combines the advantages of the exponential, Weibull, and exponential WDs, offering greater flexibility in fitting data and broader applicability, thereby enabling the analysis of more complex datasets. Tu et al. [8] introduced a Weighted Sine-generalized IWD by integrating the generalized IWD with a sine-generated probability framework. This distribution exhibits enhanced flexibility and provides a better fit compared to competing models. Zhu et al. [9] proposed a novel Sine FWD by integrating the traditional WD with a sine function. Compared to the standard Weibull, generalized Weibull, and other competing models, the Sine FWD exhibits superior fitting performance in reliability engineering and related applications. These studies have shown that by modifying the classical probability distribution model, new models can be obtained that are more in line with the characteristics of the actual data, leading to more accurate and reliable results in the field of statistical modeling and data analysis.

The inverse WD (IWD) is a probability distribution extended by WD. Compared with WD, IWD has higher flexibility and is especially suitable for describing the early failure of products. Its failure rate function can present various forms and adapt to different failure modes. This distribution can be applied to a wider set of data, especially atypical failure modes, and can effectively model the reliability of complex systems. In addition, parameter estimates for IWD may be more robust when dealing with extreme values or outliers. Therefore, it has been widely used in survival analysis, reliability analysis and life testing [10–13]. However, with the advancement of technology, we have noticed that IWD also has limitations in practical applications, especially when it comes to fitting real data, it cannot achieve the best fitting effect. Based on this, this study proposes a transformed exponential-type WD, which is a transformation based on IWD. Therefore, we name it the transformed IWD (TIWD). TIWD has a more flexible parameter structure and has a better fitting effect on real data, thereby improving the accuracy of statistical inference. In addition, the proposal of the TIWD model not only expands the existing model theory but also enriches the diversity of probability distributions in statistics. More importantly, the TIWD model also provides new ideas and methods for theoretical researchers, opening a new chapter for the research on probability distribution theory and its applications in various fields.

The rest of this article is as follows. In Section 2, we derived the mathematical expressions for the probability density function (PDF), cumulative distribution function (CDF), survival function (SF), and hazard function (HF) of the TIWD model, discussed the heavy tailed characteristics of the TIWD model, and presented relevant images of the model. In Section 3, we further analyzed the mathematical properties of the TIWD model, such as mixed representation, moments, and quantile functions. In Section 4, we analyzed various entropy measures under the TIWD model. In Section 5, we introduced several parameter estimation methods for the TIWD model, including maximum likelihood (ML) estimation, Bayesian estimation, Anderson Darling (AD) estimation, Cramer-von-Mises (CVM) estimation, and ordinary least squares (OLS) estimation. In Section 6, we compared the performance of ML estimation and three other estimation methods in parameter estimation, mean square error (MSE), and coefficient of variation (CV) through Monte Carlo simulations. In addition, this section also separately analyzed Bayesian estimation to explore the impact of different prior distributions on the estimation results. In Section 7, we applied the TIWD model to two sets of real data for analysis, in order to verify the feasibility of the model in practice. Section 8 provides relevant conclusions, limitations of the model, and future research directions.

## 2. TIWD model

Let *X* be a random variable that follows IWD, then the PDF and CDF of IWD are:


$$g(x;\beta ,\lambda )=\beta \lambda x^{-(\beta +1)}e^{-\lambda x^{-\beta }},x>0,\beta >0,\lambda >0,$$ (1)

$$G(x;\beta ,\lambda )=e^{-\lambda x^{-\beta }},x>0,\beta >0.$$ (2)

**Fig 1:**
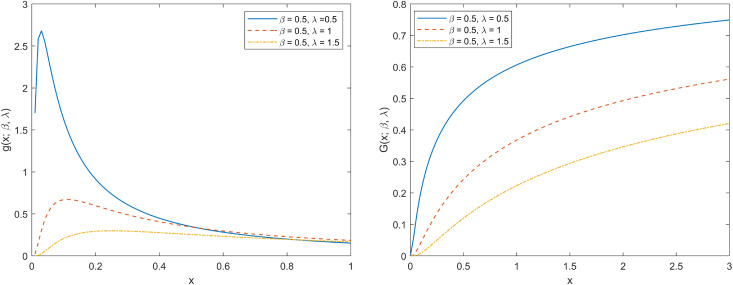
The PDF and CDF plots of IWD.

Where β is the shape parameter and λ is a known constant. As shown in Fig 1, when the parameter β is fixed, the IWD images under different λ values show significant differences. For the PDF, when 0<λ<1, the image is in an inverted bathtub shape, with a clear peak and a rapid decline after reaching the peak. When λ=1, the peak of the PDF significantly decreases, and the downward trend becomes slower. When λ>1, the PDF curve becomes more gentle. Thus, as the λ value increases, the PDF peak gradually decreases and shifts to the right, and the curve becomes smoother. For the CDF, all curves show a trend, and the larger the λ value, the more gradual the CDF increasing trend.

In this paper, we assume that λ=1. Given the transformation $F(x)=e^{\alpha (1-\frac{\text{1}}{G(x)})}$, where G(x) represents the CDF of the probability distribution model, the PDF and CDF of the TIWD are shown as follows:


$$f(x;\alpha ,\beta )=\alpha \beta x^{-\beta -1}e^{\alpha (1-e^{x^{-\beta }})}e^{x^{-\beta }},x>0,\alpha ,\beta >0,$$ (3)

$$F(x;\alpha ,\beta )=e^{\alpha (1-e^{x^{-\beta }})},x>0,\alpha ,\beta >0.$$ (4)

**Fig 2:**
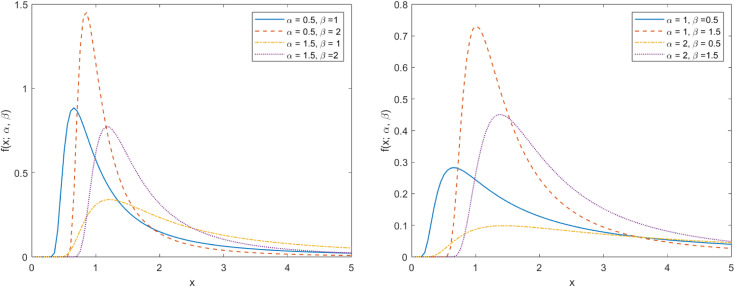
PDF plot of TIWD.

When one of the parameters is fixed, the PDF image of TIWD shows different trends as the other parameter continues to change, as shown in Fig 2. From the image, it can be intuitively seen that the PDF of the TIWD model shows a decreasing trend with the change of the random variable *X* within different parameter ranges, and the decay rate gradually slows down. To prove whether the tail of the TIWD model has heavy tail properties, we need to further investigate. We give theorem 1 to prove the heavy tail property of TIWD model.

**Theorem 1.** *Let X be a random variable following TIWD, and the distribution function* F(x) *of TIWD is heavy tailed, if for any* t>0*, the tail probability satisfies:*


$$\underset{x\to \infty }{\lim}e^{tx}P(X>x)=\infty ,$$

*where* F(x)=p(X≤x)*,* 1−F(x)=P(X>x)*.*

**Proof.** Since t>0, α,β>0, then there is $\underset{x\to \infty }{\lim}e^{tx}=\infty$. Since $\underset{x\to \infty }{\lim}e^{x^{-\beta }}=1$, therefore


$$\underset{x\to \infty }{\lim}(1-e^{\alpha (1-e^{x^{-\beta }})})=0.$$

Obviously, this is an infinitive of form 0×∞, and $\underset{x\to \infty }{\lim}\frac{\text{1}}{e^{tx}}=0$, so we rewrite $\underset{x\to \infty }{\lim}e^{tx}P(X>x)$ into the Equation (5):


$$\underset{x\to \infty }{\lim}e^{tx}P(X>x)=\underset{x\to \infty }{\lim}\frac{P(X>x)}{1/e^{tx}}$$ (5)

The Equation (5) then becomes an undetermined form of $\underset{x\to \infty }{\lim}\frac{\text{0}}{\text{0}}$, which we can use for the L’Hospital’s rule solution limitvalue. Derive the numerator and denominator in Equation (5) separately to obtain:


$$\frac{[P(X>x){]}^{\prime }}{{\left[1/e^{tx}\right]}^{\prime }}=\frac{\alpha \beta x^{-\beta -1}e^{x^{-\beta }}e^{\alpha (1-e^{x^{-\beta }})}}{t/e^{tx}}=\frac{\alpha \beta e^{x^{-\beta }}e^{\alpha (1-e^{x^{-\beta }})}e^{tx}}{tx^{\beta +1}}$$

Then,


$$\underset{x\to \infty }{\lim}\frac{\alpha \beta e^{x^{-\beta }}e^{\alpha (1-e^{x^{-\beta }})}e^{tx}}{tx^{\beta +1}}=\underset{x\to \infty }{\lim}\frac{\alpha \beta e^{tx}}{tx^{\beta +1}}$$ (6)

Continuing the L’Hospital’s rule limit on Equation (6), we obtain:


$$\frac{(\alpha \beta e^{tx}{)}^{\prime }}{(tx^{\beta +1}{)}^{\prime }}=\frac{\alpha \beta e^{tx}}{(\beta +1)x^{\beta }}$$

By doing Lopida over and over again, we get:


$$\underset{x\to \infty }{\lim}e^{tx}(1-e^{\alpha (1-e^{x^{-\beta }})})=\infty$$

Therefore, by combining the intuitive judgment of PDF images with the quantitative analysis of the tail probability of CDF, we can comprehensively evaluate and confirm the heavy-tailed characteristic of the TIWD model. As shown in Fig 2, the right tail decay speed of the TIWD model is slower than that of the IWD model. Moreover, as the β value increases, all curves show a trend of becoming more concentrated, which means they all have light-tailed characteristics. In other words, a high β value leads to a shorter tail of the distribution, while a low β value may result in a longer tail or a heavy-tailed phenomenon.

The SF and HF of TIWD are:


$$S(x;\alpha ,\beta )=1-F(x;\alpha ,\beta )=1-e^{\alpha (1-e^{x^{-\beta }})},$$ (7)

$$h(x;\alpha ,\beta )=\frac{f(x;\alpha ,\beta )}{S(x;\alpha ,\beta )}=\frac{\alpha \beta x^{-\beta -1}e^{\alpha (1-e^{x^{-\beta }})}e^{x^{-\beta }}}{1-e^{\alpha (1-e^{x^{-\beta }})}}$$ (8)

**Fig 3:**
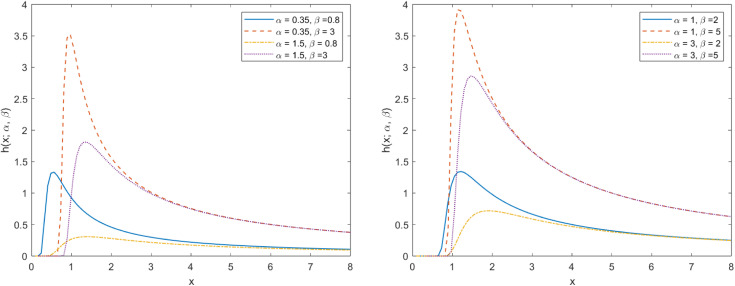
HF plot of TIWD.

Fig 3 shows the HF curves of the TIWD model under various parameter settings. When observing the image, it is evident that when one parameter remains constant, the HF image exhibits an inverted bathtub shape as the other parameter changes. This indicates that the stability and reliability of the product or system improve over time. In addition, it also indicates that compared to classical models, the model can be adapted to different risk control requirements by setting different parameters, enhancing its significant advantages in adaptability and flexibility. When further analyzing the HF curve, we observed that as the independent variable *x* increases, the curve as a whole tends to approach zero. This phenomenon indicates that the HF curve displays distinct heavy tailed features. In other words, the curve shows a slower decay rate in areas with higher *X* values, reflecting a thicker tail than the light tail distribution. The heavy tail characteristic can effectively avoid risk assessment under light tail distribution, thereby ensuring the predictability of risks in high reliability scenarios.

The TIWD shares certain similarities with the inverse Chen distribution. Both are derived through mathematical transformations of their original distributions, and their PDF and HF curves exhibit similar morphological characteristics, demonstrating heavy-tailed features within specific parameter ranges. However, their key difference lies in their transformation mechanisms. The TIWD is obtained by introducing additional parameters for mathematical transformation while keeping one parameter of the original distribution fixed [14], whereas the inverse Chen distribution relies solely on the parameters of the original distribution for its transformation.

Cumulative HF and inverse HF are two important concepts in reliability analysis, closely related to HF. The cumulative HF represents the integral of HF from the initial moment to time *t*, which is the cumulative amount of failure risk within that time interval. At the same time, the cumulative HF is closely related to SF, which represents the probability that the product has not failed before time *t*. The reverse HF is used to analyze the probability of failure after time *t*, and can be used to evaluate the risk of product failure during the remaining life. Here we give the expressions of these two functions [15]:


$$H(x;\alpha ,\beta )=\int_{0}^{t}h(x;\alpha ,\beta )dx=-\ln[S(x;\alpha ,\beta )]=-\ln[1-e^{\alpha (1-e^{x^{-\beta }})}],$$

$$r(x;\alpha ,\beta )=\frac{f(x;\alpha ,\beta )}{F(x;\alpha ,\beta )}=\alpha \beta x^{-\beta -1}e^{x^{-\beta }}$$

## 3. Mathematical properties

### 3.1. Moments

The *r*-th moment of the TIWD model can be computed utilizing the subsequent mathematical expression:


$$\begin{array}{cc} {\mu_{r}}^{\prime }=E[X^{r}] & =\int_{0}^{\infty }x^{r}f(x;\alpha ,\beta )dx\\ & =r\int_{0}^{\infty }x^{r-1}S(x;\alpha ,\beta )dx \end{array}$$ (9)

According to Equation (3), when x→∞, the exponential function can be expanded by Taylor to obtain $S(x)\sim \alpha x^{-\beta }$. Therefore


$$\underset{x\to \infty }{\lim}\frac{S(x)}{\alpha x^{-\beta }}=1,$$

then *X* is said to have a power-law distribution. For the power-law distribution $S(x)\sim \alpha x^{-\beta }$, the existence of its *r*-order moment $E[X^{r}]$ is related to β, that is, when x→∞, there is


$$\begin{array}{c} {\mu_{r}}^{\prime }=E[X^{r}]\sim r\alpha \int_{0}^{\infty }x^{r-1-\beta }dx \end{array}$$

According to the necessary and sufficient condition for integral convergence, there is


r−1−β<−1⇒r<β.

Therefore, the *r*-order moment is finite if and only if r<β.

By substituting Equation (3) into Equation (9), we get:


$$\begin{array}{c} \begin{array}{c} {\mu_{r}}^{\prime }=\int_{0}^{\infty }x^{r}\alpha \beta x^{-\beta -1}e^{\alpha (1-e^{x^{-\beta }})}e^{x^{-\beta }}dx\\ =\int_{0}^{\infty }\alpha \beta x^{r-\beta -1}e^{\alpha (1-e^{x^{-\beta }})}e^{x^{-\beta }}dx. \end{array} \end{array}$$ (10)

Let $e^{x^{-\beta }}=t$, then Equation (10) is simplified as:


$$\begin{array}{c} {\mu_{r}}^{\prime }=\int_{1}^{\infty }\alpha {\left(\log t\right)}^{-\frac{r}{\beta }}e^{\alpha (1-t)}dt \end{array}$$ (11)

Therefore, from Equation (11), we can see that the mean and variance of *X* are:


$$\begin{array}{c} \mu =\int_{1}^{\infty }\alpha {\left(\log t\right)}^{-\frac{\text{1}}{\beta }}e^{\alpha (1-t)}dt, \end{array}$$ (12)

$$\begin{array}{c} \sigma^{2}=\int_{1}^{\infty }\alpha {\left(\log t\right)}^{-\frac{\text{2}}{\beta }}e^{\alpha (1-t)}dt-(\int_{\infty }^{1}\alpha {\left(\log t\right)}^{-\frac{\text{1}}{\beta }}e^{\alpha (1-t)}dt{)}^{2} \end{array}$$ (13)

### 3.2. Incomplete moments

Incomplete moments, as an important statistical measure for describing the partial order moments of random variables, play a crucial role in fields such as risk management, financial mathematics, and extreme value theory, especially in the assessment of extreme event risks [16]. In the TIWD model, indepth analysis of the characteristics of incomplete moments will provide us with valuable insights into the distribution characteristics of random variables and expand their potential applications in multiple fields. The definition of incomplete *r*-th moment for continuous random variable *X* and its PDF is as follows [17]:


$$\begin{array}{c} I_{r}(x)=\int_{0}^{x}s^{r}f(s;\alpha ,\beta )ds \end{array}$$ (14)

Therefore, substitute Equation (3) into Equation (14) to obtain the *r*-th incomplete moment of the random variable *X*:


$$\begin{array}{c} I_{r}(x)=\int_{0}^{x}s^{r}\alpha \beta s^{-\beta -1}e^{\alpha (1-e^{s^{-\beta }})}e^{s^{-\beta }}ds\\ =\int_{e^{x^{-\beta }}}^{\infty }\alpha {\left(\log v\right)}^{-\frac{r}{\beta }}e^{\alpha (1-v)}dv. \end{array}$$ (15)

The Lorenz curve is a tool in economics used to describe the degree of inequality in income or wealth distribution. It visualizes the uniformity of income and wealth distribution through images. In statistics, incomplete moments are an important tool used to describe the characteristics of income and wealth distribution. Although the two differ in terms of imagery and mathematical expressions, they both analyze economic distribution characteristics from different perspectives. The corresponding expression is given by the formula for the Lorenz curve below [18]:


$$\begin{array}{c} L(x)=\frac{\text{1}}{\mu }\int_{0}^{x}tf(t)dt=\frac{I_{1}(x)}{\mu }\\ =\frac{\text{1}}{\mu }\int_{e^{x^{-\beta }}}^{\infty }\alpha {\left(\log v\right)}^{-\frac{\text{1}}{\beta }}e^{\alpha (1-v)}dv. \end{array}$$ (16)

The Bonferroni curve, as a statistical method, is primarily used for multiple hypothesis testing and is defined as the ratio of the Lorenz curve to the CDF [19]:


$$\begin{array}{c} B(x)=\frac{L(x)}{F(x;\alpha ,\beta )}\\ =\frac{\int_{e^{x^{-\beta }}}^{\infty }\alpha {\left(\log v\right)}^{-\frac{\text{1}}{\beta }}e^{\alpha (1-v)}dv}{\mu e^{\alpha (1-e^{x^{-\beta }})}}. \end{array}$$ (17)

### 3.3 Quantile function

Quantile functions, serving as a pivotal instrument in the realms of statistics and probability theory, facilitate the comprehension and analytical dissection of data attributes. Furthermore, they are instrumental in diverse statistical inferential and decision-making frameworks. These functions can be derived through the process of inverse transformation applied to the CDF:


$$\rho (w)=[F(x;\alpha ,\beta ){]}^{-1}=[\ln(1-\frac{\text{1}}{\alpha }\ln w){]}^{-\frac{\text{1}}{\beta }},0<w<1.$$

**Fig 4:**
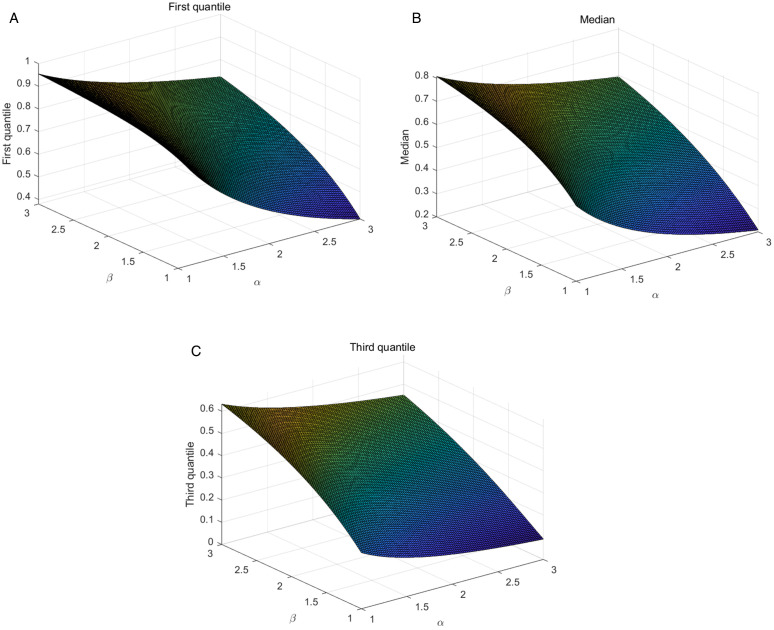
3D surface plots of the first quartile, median and the third quartile.

**Fig 5:**
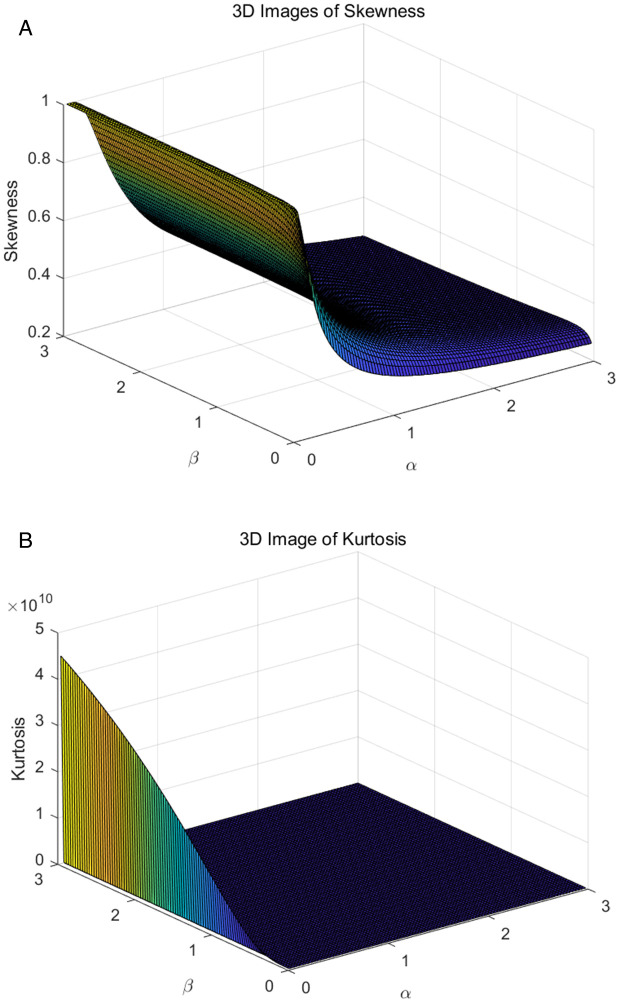
3D surface plots of skewness and kurtosis under different parameter ranges.


Among them, *w* represents the probability value, ranging from 0 to 1. When w equals 0.25, 0.5, and 0.75, they correspond to the first quartile, median and the third quartile, respectively. In Fig 4, we present 3D plots of the first quartile, median and the third quartile. From these plots, we can observe that as the probability values change, the degree of skewness in the distribution gradually becomes smoother. Skewness and kurtosis are important statistical measures used to characterize the state of data distribution. In Fig 5, we present plots of the skewness and kurtosis of the TIWD model across different parameter ranges. We will provide the expressions for skewness and kurtosis:

$$S=\frac{p(\frac{\text{1}}{\text{4}})+p(\frac{\text{3}}{\text{4}})-2p(\frac{\text{1}}{\text{2}})}{p(\frac{\text{3}}{\text{4}})-p(\frac{\text{1}}{\text{4}})},K=\frac{p(\frac{\text{7}}{\text{8}})-p(\frac{\text{5}}{\text{8}})+p(\frac{\text{3}}{\text{8}})-p(\frac{\text{1}}{\text{8}})}{p(\frac{\text{6}}{\text{8}})-p(\frac{\text{2}}{\text{8}})}.$$

### 3.4 Order statistics

Order statistics are a powerful and versatile tool in statistics, with extensive applications in data analysis, optimization problems, decision-making, and theoretical research. Let $X_{1},X_{2},\cdots ,X_{n}$ be random samples from the total sample *X*, where $x_{1},x_{2},\cdots ,x_{n}$ are observations of the random samples. Arrange samples $X_{1},X_{2},\cdots ,X_{n}$ in ascending order to obtain $X_{\left(1\right)},X_{\left(2\right)},\cdots ,X_{\left(n\right)}$, then it is called $X_{\left(1\right)},X_{\left(2\right)},\cdots ,X_{\left(n\right)}$ is the order statistic of $X_{1},X_{2},\cdots ,X_{n}$, where $X_{\left(1\right)}$ is the minimum order statistic of the sample and $X_{\left(n\right)}$ is the maximum order statistic of the sample. From Equations (3) and (4), we can obtain the distribution density of the *j*-th order statistic $X_{\left(j\right)}$ of TIWD, which is:


$$\begin{array}{c} f_{\left(j\right)}(x;\alpha ,\beta )=\frac{n!}{(j-1)!(n-j)!}f(x)[F(x){]}^{j-1}[1-F(x){]}^{n-j}\\ =\frac{n!}{(j-1)!(n-j)!}\alpha \beta x^{-\beta -1}e^{\alpha (1-e^{x^{-\beta }})}e^{x^{-\beta }}e^{\alpha (1-e^{x^{-\beta }})(j-1)}[1-e^{\alpha (1-e^{x^{-\beta }})}{]}^{n-j}\\ =\frac{n!}{(j-1)!(n-j)!}\alpha \beta x^{-\beta -1}e^{\alpha (1-e^{x^{-\beta }})j+x^{-\beta }}[1-e^{\alpha (1-e^{x^{-\beta }})}{]}^{n-j}. \end{array}$$ (18)

So the minimum order statistic is:


$$f_{\left(1\right)}(x;\alpha ,\beta )=n\alpha \beta x^{-\beta -1}e^{\alpha (1-e^{x^{-\beta }})+x^{-\beta }}[1-e^{\alpha (1-e^{x^{-\beta }})}{]}^{n-1}.$$

The maximum order statistic is:


$$f_{\left(n\right)}(x;\alpha ,\beta )=n\alpha \beta x^{-\beta -1}e^{\alpha (1-e^{x^{-\beta }})n+x^{-\beta }}.$$

### 3.5 Mean-residual life

The mean-residual life is an important concept in reliability analysis and product life testing. It refers to the average time the system can continue to operate correctly after a specific point in time *t*, which can be calculated using the probability density of the remaining life. The corresponding calculation formula is as follows [20]:


$$\begin{array}{c} m(x)=E[\left(X-x\right)|X>x]=\frac{\int_{x}^{\infty }S(y)dy}{S(x)}\\ =\frac{\text{1}}{S(x)}\int_{x}^{\infty }[1-e^{\alpha (1-e^{y^{-\beta }})}]dy. \end{array}$$

Among them, S(x) is the SF, which can be known from Equation (7).

## 4. Entropy measure under TIWD model

### 4.1. Shannon entropy

Shannon entropy, a core concept in information theory, quantifies the degree of uncertainty in information and plays a crucial role in various fields, including cryptography, information coding, and communication systems. In statistics, Shannon entropy evaluates probability models, providing rigorous theoretical support for data analysis and model construction, thereby enhancing the depth and accuracy of data analysis. The expression of Shannon entropy within the TIWD model is delineated as follows [21]:


$$\begin{array}{c} H_{S}=-\int_{-\infty }^{+\infty }f(x;\alpha ,\beta )\ln[f(x;\alpha ,\beta )]dx\\ =-\int_{0}^{+\infty }f(x;\alpha ,\beta )[\ln(\alpha \beta )-(\beta +1)\ln x+\alpha (1-e^{x^{-\beta }})+x^{-\beta }]dx\\ =-\ln(\alpha \beta )+(\beta +1)E[\ln X]-\alpha E[1-e^{X^{-\beta }}]-E[X^{-\beta }]. \end{array}$$ (19)

### 4.2. Rényi entropy

As an extension of Shannon entropy, Rényi entropy shows its strong flexibility through the introduction of parameters ξ, so it has a wide range of applications in machine learning, image processing, bioinformatics and other fields [22–25]. The expression of Rényi entropy within the TIWD model is delineated as follows:


$$\begin{array}{c} H_{R}=\frac{\text{1}}{1-\xi }\log[\int_{0}^{\infty }f^{\xi }(x;\alpha ,\beta )dx],\xi \neq 1,\xi >0\\ =\frac{\text{1}}{1-\xi }\log\{\int_{0}^{\infty }[\alpha \beta x^{-\beta -1}e^{\alpha (1-e^{x^{-\beta }})}e^{x^{-\beta }}{]}^{\xi }dx\}\\ =\frac{\text{1}}{1-\xi }\log\{\int_{0}^{\infty }{\left(\alpha \beta \right)}^{\xi }x^{\xi (-\beta -1)}e^{\xi \alpha (1-e^{x^{-\beta }})}e^{\xi x^{-\beta }}dx\}. \end{array}$$ (20)

### 4.3. Tsallis entropy

Tsallis entropy, conceptualized as a generalization of entropy, was introduced by Constantino Tsallis in 1988. Through the incorporation of the entropy parameter δ, Tsallis entropy offers a more robust theoretical framework and pragmatic approach for scaling analysis, optimal decision-making, and machine learning, thereby enhancing the modeling of real-world complexities and diversities. The expression of Tsallis entropy within the TIWD model is delineated as follows [21]:


$$\begin{array}{c} H_{T}=\frac{\text{1}}{1-\delta }[\int_{\text{0}}^{\infty }f^{\delta }(x;\alpha ,\beta )dx-1],\delta \neq 1,\delta <0\\ =\frac{\text{1}}{1-\delta }\left\{\int_{\text{0}}^{\infty }{\left[\alpha \beta x^{-\beta -1}e^{\alpha (1-e^{x^{-\beta }})}e^{x^{-\beta }}\right]}^{\delta }dx-1\right\}. \end{array}$$ (21)

### 4.4. Mathai–Haubold entropy

The Mathai-Haubold entropy, advanced within the domain of statistical mechanics, offers a more nuanced representation of certain system configurations. Its distinct mathematical attributes and utility in the context of complex systems render the Mathai-Haubold entropy a pivotal construct in the disciplines of mathematics and physics. The expression of Mathai–Haubold entropy within the TIWD model is delineated as follows [26]:


$$\begin{array}{c} H_{M}=\frac{\text{1}}{\varsigma -1}[\int_{\text{0}}^{\infty }f^{\left(2-\varsigma \right)}(x;\alpha ,\beta )dx-1],\varsigma \neq 1,0<\varsigma <2\\ =\frac{\text{1}}{\varsigma -1}\left\{\int_{\text{0}}^{\infty }{\left[\alpha \beta x^{-\beta -1}e^{\alpha (1-e^{x^{-\beta }})}e^{x^{-\beta }}\right]}^{2-\varsigma }dx-1\right\}. \end{array}$$ (22)

## 5. Parameter estimation of TIWD model

### 5.1. ML estimation

Let $x_{1},x_{2},\cdots ,x_{n}$ is the n sample size from the TIWD model, denoted $x^{*}=(x_{1},x_{2},\cdots ,x_{n})$, and the likelihood function (LF) can be defined as follows:


$$L(\alpha ,\beta |x^{*})=\prod_{i=1}^{n}f(x_{i};\alpha ,\beta )=(\alpha \beta {)}^{n}\prod_{i=1}^{n}x_{i}^{-\beta -1}e^{\alpha (1-e^{x_{i}^{-\beta }})}e^{x_{i}^{-\beta }}.$$ (23)

The log-LF is then:


$$l(\alpha ,\beta |x^{*})=n\ln(\alpha \beta )-(\beta +1)\sum {\mathrm{\backslash nolimits}}_{i=1}^{n}\ln x_{i}+\alpha \sum {\mathrm{\backslash nolimits}}_{i=1}^{n}\left(1-e^{x_{i}^{-\beta }}\right)+\sum {\mathrm{\backslash nolimits}}_{i=1}^{n}x_{i}^{-\beta }.$$ (24)

Subsequently, we derive the parameters in Equation (24) individually to elucidate the likelihood Equations (25) and (26):


$$\frac{\partial l(\alpha ,\beta |x^{*})}{\partial \alpha }=\frac{n}{\alpha }+\sum {\mathrm{\backslash nolimits}}_{i=1}^{n}(1-e^{x_{i}^{-\beta }}),$$ (25)

$$\frac{\partial l(\alpha ,\beta |x^{*})}{\partial \beta }=\frac{n}{\beta }-\sum {\mathrm{\backslash nolimits}}_{i=1}^{n}\ln x_{i}+\alpha \sum {\mathrm{\backslash nolimits}}_{i=1}^{n}e^{x_{i}^{-\beta }}x_{i}^{-\beta }\ln x_{i}-\sum {\mathrm{\backslash nolimits}}_{i=1}^{n}x_{i}^{-\beta }\ln x_{i}.$$ (26)

ML estimates for the parameters α and β can be derived by resolving the Equations (25) and (26). However, direct resolution of these equations is computationally complex, necessitating the use of numerical computation to ascertain the parameters within them. In this study, we employ the dichotomy method to numerically calculate the parameters and obtain the ML estimates $\hat{\alpha }$ and $\hat{\beta }$ for the parameters α and β. To ensure the reliability and effectiveness of the ML estimation, we need to demonstrate the existence and uniqueness of the ML estimate. This process not only ensures the accuracy of statistical inference, but also enhances the application value of the ML estimation in practical data analysis and model parameter estimation.

#### 5.1.1 Existence and uniqueness of ML estimation solutions.

**Theorem 2. ***Let the left side of Equation* (25)* be *$p(\alpha )=\frac{n}{\alpha }+\sum {\mathrm{\backslash nolimits}}_{i=1}^{n}\left(1-e^{x_{i}^{-\beta }}\right)$*, then the solution* $\hat{\alpha }$ *obtained by* p(α)=0 *exists and is unique on* (0,+∞)*.*

**Proof.** (1) Existence

When α→0, it is obvious that $\underset{\alpha \to 0}{\lim}\frac{n}{\alpha }=+\infty$, so $\underset{\alpha \to 0}{\lim}p(\alpha )=+\infty .$

When α→+∞, we have $\underset{\alpha \to +\infty }{\lim}\frac{n}{\alpha }=0$, so $\underset{\alpha \to +\infty }{\lim}p(\alpha )=\sum {\mathrm{\backslash nolimits}}_{i=1}^{n}\left(1-e^{x_{i}^{-\beta }}\right).$

Due to x, α, β>0, then $e^{x^{-\beta }}>1$, $1-e^{x_{i}^{-\beta }}<0$, then $\underset{\alpha \to +\infty }{\lim}p(\alpha )<0$.

Since p(α) is continuous on (0,+∞), the intermediate value theorem shows that p(α) has at least one solution $\hat{\alpha }$ on (0,+∞) such that $p(\hat{\alpha })=0$, proving existence.

(2) Uniqueness

Taking the derivative of α in p(α) gives the following formula:

$p^{\prime }(\alpha )=-\frac{n}{\alpha^{2}}$.

It is obvious that $p^{\prime }(\alpha )<0$, so we can see that p(α) is a monotone decreasing function. If a strictly monotonically decreasing function intersects with the *x*-axis, there is at most one intersection point. Combining ‘existence’, it can be inferred that there exists at least one solution, thus proving uniqueness.

**Theorem 3.** *Let the left side of Equation *(26)* be*


$$p(\beta )=\frac{n}{\beta }-\sum {\mathrm{\backslash nolimits}}_{i=1}^{n}\ln x_{i}+\alpha \sum {\mathrm{\backslash nolimits}}_{i=1}^{n}e^{x_{i}^{-\beta }}x_{i}^{-\beta }\ln x_{i}-\sum {\mathrm{\backslash nolimits}}_{i=1}^{n}x_{i}^{-\beta }\ln x_{i}$$

*then the solution* $\hat{\beta }$ *obtained by* p(β)=0 *exists and is unique on* (0,+∞)*.*

**Proof.** (1) Existence

When β→0, it is obvious that

$\underset{\beta \to 0}{\lim}\frac{n}{\beta }=+\infty$, $\underset{\beta \to 0}{\lim}\alpha \sum {\mathrm{\backslash nolimits}}_{i=1}^{n}e^{x_{i}^{-\beta }}x_{i}^{-\beta }\ln x_{i}=\alpha \sum {\mathrm{\backslash nolimits}}_{i=1}^{n}e\ln x_{i}$, $\underset{\beta \to 0}{\lim}\sum {\mathrm{\backslash nolimits}}_{i=1}^{n}x_{i}^{-\beta }\ln x_{i}=\sum {\mathrm{\backslash nolimits}}_{i=1}^{n}\ln x_{i}$,

So $\underset{\beta \to 0}{\lim}p(\beta )=+\infty$.

When β→+∞, we have $\underset{\beta \to +\infty }{\lim}\frac{n}{\beta }=0$.

Also because $\underset{\beta \to +\infty }{\lim}\alpha \sum {\mathrm{\backslash nolimits}}_{i=1}^{n}e^{x_{i}^{-\beta }}x_{i}^{-\beta }\log x_{i}$ and $\underset{\beta \to +\infty }{\lim}\sum {\mathrm{\backslash nolimits}}_{i=1}^{n}x_{i}^{-\beta }\log x_{i}$ are related to the values of *x*, the following discussion now follows:

When x<1, $x^{-\beta }\to 0$, $e^{x^{-\beta }}\to 1$, $e^{x^{-\beta }}x^{-\beta }\log x\to 0$, $x^{-\beta }\log x\to 0$,

so $\lim_{\beta \to +\infty }p(\beta )=-\sum_{i=1}^{n}\log x_{i}<0$.

When x=1, $x^{-\beta }=1$, $e^{x^{-\beta }}=e$, $e^{x^{-\beta }}x^{-\beta }\log x=0$, $x^{-\beta }\log x=0$,

so $\underset{\beta \to +\infty }{\lim}p(\beta )=-\sum {\mathrm{\backslash nolimits}}_{i=1}^{n}\log x_{i}=0$.

When 0<x<1, $x^{-\beta }\to +\infty$, $e^{x^{-\beta }}\to +\infty$, $e^{x^{-\beta }}x^{-\beta }\log x\to -\infty$, $-x^{-\beta }\log x\to +\infty$,

so we need to compare the growth rates of $e^{x^{-\beta }}x^{-\beta }\log x\to -\infty$ and $-x^{-\beta }\log x\to +\infty$.

Let $t=x^{-\beta }$, logx=−c(c(0), so we have

$\alpha e^{x^{-\beta }}x^{-\beta }\log x=\alpha e^{t}t(-c)=-\alpha e^{t}tc$, $-x^{-\beta }\log x=-t(-c)=tc$.

Thus $-\alpha e^{t}tc+tc=tc(1-\alpha e^{t})$. Because the growth rate of $e^{t}$ is faster than any other term and $\alpha e^{t}\to +\infty$, $tc(1-\alpha e^{t})\to -\infty$. Therefore, there exists at least one 0<x<1 with $\lim_{\beta \to +\infty }p(\beta )<0$.

In summary, when β→+∞, there exists at least one solution with $\lim_{\beta \to +\infty }p(\beta )<0$ for 0<x<1, and $\lim_{\beta \to +\infty }p(\beta )<0$ for all x≥1. Therefore, according to the intermediate value theorem, it can be concluded that p(β) has at least one solution $\hat{\beta }$ on (0,+∞) such that $p(\hat{\beta })=0$, proving existence.

(2) Uniqueness

The derivative of β in p(β) yields the following equation:


$$p^{\prime }(\beta )=-\frac{n}{\beta^{2}}-\alpha \sum {\mathrm{\backslash nolimits}}_{i=1}^{n}x_{i}^{-\beta }{\left(\ln x_{i}\right)}^{2}e^{x_{i}^{-\beta }}(x_{i}^{-\beta }+1)+\sum {\mathrm{\backslash nolimits}}_{i=1}^{n}x_{i}^{-\beta }(\ln x_{i}{)}^{2}$$ (27)

Given that $-\frac{n}{\beta^{2}}<0$, to verify that $p^{\prime }(\beta )<0$, it is necessary to compare the magnitudes of the second and third terms. Given that $x^{-\beta }>0$ and $e^{x^{-\beta }}>1$, it follows that


$$\alpha x^{-\beta }(\log x{)}^{2}e^{x^{-\beta }}(x^{-\beta }+1)>\alpha x^{-\beta }(\log x{)}^{2}1\cdot 1=\alpha x^{-\beta }(\log x{)}^{2}$$

If α≥1, then


$$\alpha x^{-\beta }(\log x{)}^{2}e^{x^{-\beta }}(x^{-\beta }+1)>x^{-\beta }(\log x{)}^{2}$$

If 0<α<1, the growth rate of $e^{x^{-\beta }}$ is faster than the reduction rate of α. At this time, the inequality


$$\alpha x^{-\beta }(\log x{)}^{2}e^{x^{-\beta }}(x^{-\beta }+1)>x^{-\beta }(\log x{)}^{2}$$

still holds. So we have


$$\alpha \sum {\mathrm{\backslash nolimits}}_{i=1}^{n}x_{i}^{-\beta }{\left(\log x_{i}\right)}^{2}e^{x_{i}^{-\beta }}(x_{i}^{-\beta }+1)>\sum {\mathrm{\backslash nolimits}}_{i=1}^{n}x_{i}^{-\beta }{\left(\log x_{i}\right)}^{2}$$

Which implies


$$-\alpha \sum {\mathrm{\backslash nolimits}}_{i=1}^{n}x_{i}^{-\beta }{\left(\log x_{i}\right)}^{2}e^{x_{i}^{-\beta }}(x_{i}^{-\beta }+1)<-\sum {\mathrm{\backslash nolimits}}_{i=1}^{n}x_{i}^{-\beta }{\left(\log x_{i}\right)}^{2}$$

Thus,


$$\begin{array}{c} p^{\prime }(\beta )=-\frac{n}{\beta^{2}}+\left(-\alpha \sum {\mathrm{\backslash nolimits}}_{i=1}^{n}x_{i}^{-\beta }{\left(\log x_{i}\right)}^{2}e^{x_{i}^{-\beta }}(x_{i}^{-\beta }+1)\right)+\sum {\mathrm{\backslash nolimits}}_{i=1}^{n}x_{i}^{-\beta }(\log x_{i}{)}^{2}<0 \end{array}$$

Since $p^{\prime }(\beta )<0$, it follows that p(β) is a monotonically decreasing function on (0,+∞). Combining with the “existence” proof, we know that there is at least one solution on (0,+∞), so we have proved the uniqueness. ☐

#### 5.1.2 Asymptotic confidence interval.

In this section, we construct asymptotic confidence intervals (ACIs) for the parameters to evaluate the performance of ML estimates in terms of precision, stability, and applicability. Since ML estimates are asymptotically normal, we can use $Var(\hat{\alpha })$ and $Var(\hat{\beta })$ to construct ACIs for α and β. The asymptotic variance needs to be obtained from the inverse of the Fisher information matrix, which is the negative value of the Hessian matrix of the log-LF, i.e.,


$$I(\alpha ,\beta )=\left[\begin{array}{cc} {}*20c-\frac{\partial^{2}l(\alpha ,\beta |x^{*})}{\partial \alpha^{2}} & -\frac{\partial^{2}l(\alpha ,\beta |x^{*})}{\partial \alpha \partial \beta }\\ -\frac{\partial^{2}l(\alpha ,\beta |x^{*})}{\partial \beta \partial \alpha } & -\frac{\partial^{2}l(\alpha ,\beta |x^{*})}{\partial \beta^{2}} \end{array}\right].$$

Where


$$\frac{\partial^{2}l(\alpha ,\beta |x^{*})}{\partial \alpha^{2}}=-\frac{n}{\alpha^{2}}$$

$$\frac{\partial^{2}l(\alpha ,\beta |x^{*})}{\partial \alpha \partial \beta }=\sum {\mathrm{\backslash nolimits}}_{i=1}^{n}e^{x_{i}^{-\beta }}x_{i}^{-\beta }\ln x_{i}=\frac{\partial^{2}l(\alpha ,\beta |x^{*})}{\partial \beta \partial \alpha }$$

$$\frac{\partial^{2}l(\alpha ,\beta |x^{*})}{\partial \beta^{2}}=-\frac{n}{\beta^{2}}-\alpha \sum {\mathrm{\backslash nolimits}}_{i=1}^{n}e^{x_{i}^{-\beta }}x_{i}^{-\beta }{\left(\ln x_{i}\right)}^{2}(x_{i}^{-\beta }+1)+\sum {\mathrm{\backslash nolimits}}_{i=1}^{n}x_{i}^{-\beta }{\left(\ln x_{i}\right)}^{2}.$$

We substitute the obtained ML estimates $\hat{\alpha }$ and $\hat{\beta }$ into the Fisher information moment to obtain:


$$I(\hat{\alpha },\hat{\beta })={\left[\begin{array}{cc} {}*20c-\frac{\partial^{2}l(\alpha ,\beta |x^{*})}{\partial \alpha^{2}} & -\frac{\partial^{2}l(\alpha ,\beta |x^{*})}{\partial \alpha \partial \beta }\\ -\frac{\partial^{2}l(\alpha ,\beta |x^{*})}{\partial \beta \partial \alpha } & -\frac{\partial^{2}l(\alpha ,\beta |x^{*})}{\partial \beta^{2}} \end{array}\right]}_{\alpha =\hat{\alpha },\beta =\hat{\beta }}.$$

Then the covariance matrix of $\hat{\alpha }$ and $\hat{\beta }$ is:


$$I^{-1}(\hat{\alpha },\hat{\beta })=\left[\begin{array}{cc} {}*20c\hat{V}ar(\hat{\alpha }) & \hat{C}ov(\hat{\alpha },\hat{\beta })\\ \hat{C}ov(\hat{\beta },\hat{\alpha }) & \hat{V}ar(\hat{\beta }) \end{array}\right].$$

The 100(1−θ ACIs for α and β is:

$\hat{\alpha }\pm Z_{\theta /2}\sqrt{\hat{V}ar(\hat{\alpha })}$, $\hat{\beta }\pm Z_{\theta /2}\sqrt{\hat{V}ar(\hat{\beta })}$.

Where $Z_{\theta /2}$ is the θ/2 percentile of the standard normal distribution.

### 5.2 Bayesian estimation

Bayesian estimation is a statistical inference method based on Bayes’ theorem, which is based on the principle of combining a priori knowledge with observed data to obtain estimates of parameters [27]. In this process, prior knowledge plays an important role in Bayesian estimation, which not only provides the basis for data analysis, but also determines the posterior distribution of the parameters in combination with the observed data. Therefore, choosing a reasonable prior distribution is decisive for ensuring the accuracy, reliability and stability of parameter estimation. As the conjugate prior of Poisson and exponential distributions, the gamma distribution can flexibly express different prior information by adjusting the parameter values, thus simplifying the computational process of Bayesian estimation and improving the accuracy and reliability of the estimation results. Therefore, in this paper, we choose the gamma distribution as the prior distribution of α and β. Assuming that α and β are independent random variables and follow $\Gamma (\eta_{1},\psi_{1})$ and $\Gamma (\eta_{2},\psi_{2})$, respectively, the joint prior distribution of α and β is:


$$\pi_{1}(\alpha ,\beta )=\frac{\psi_{1}^{\eta_{1}}\psi_{2}^{\eta_{2}}}{\Gamma (\eta_{1})\Gamma (\eta_{2})}\alpha^{\eta_{1}-1}\beta^{\eta_{2}-1}e^{-\psi_{1}\alpha -\psi_{2}\beta },\alpha ,\beta >0$$ (28)

The joint posterior density of α and β is:


$$\pi_{1}(\alpha ,\beta |x^{*})\propto \alpha^{\eta_{1}+n-1}\beta^{\eta_{2}+n-1}e^{-\psi_{1}\alpha -\psi_{2}\beta }\prod_{i=1}^{n}x_{i}^{-\beta -1}e^{\alpha (1-e^{x_{i}^{-\beta }})}e^{x_{i}^{-\beta }}$$ (29)

To evaluate the influence of prior information on the estimation results, we also consider non-informative priors in this study. By comparing the results from these two types of priors, we can verify the stability of the TIWD model. When the prior distribution of α and β is non-informative prior, then the joint prior distribution of α and β is:


$$\pi_{2}(\alpha ,\beta )=\frac{\text{1}}{\alpha \beta },\alpha ,\beta >0$$ (30)

The joint posterior density of α and β is:


$$\pi_{2}(\alpha ,\beta |x^{*})\propto \alpha^{n-1}\beta^{n-1}\prod_{i=1}^{n}x_{i}^{-\beta -1}e^{\alpha (1-e^{x_{i}^{-\beta }})}e^{x_{i}^{-\beta }}.$$ (31)

In Bayesian estimation, we usually introduce a loss function to quantify the cost loss or decision errors that occur during the estimation process, thereby transforming statistical inference into an optimization problem. In this section, we introduce the precautionary loss function (PLF) to estimate the parameters of the TIWD model. Next, we will analyze Bayesian estimation under PLF. According to Akhtar [28], the definition of PLF is:


$$L(\hat{\theta },\theta )=\frac{{\left(\hat{\theta }-\theta \right)}^{2}}{\hat{\theta }}$$

Thus, the Bayesian estimator under PLF is:


$$\hat{\varpi }(\alpha ,\beta )=\sqrt{E(\varpi |x^{*})}=\sqrt{\int_{0}^{\infty }\int_{0}^{\infty }\varpi (\alpha ,\beta )\pi (\alpha ,\beta |x^{*})d\alpha d\beta }.$$ (32)

Among them, θ=(α,β), $\hat{\theta }=(\hat{\alpha },\hat{\beta })$, and ϖ(α,β) represent functions about α and β. From Equation (32), it can be seen that the Bayesian estimator of TIWD under PLF is non explicit, and it is relatively complex to directly calculate Equation (32). Therefore, we consider using MCMC sampling to obtain the Bayesian estimates under PLF.

MCMC sampling, a foundational statistical technique, lies at the heart of its ability to efficiently draw samples from complex probability distributions. This is achieved through the integration of Markov chains and Monte Carlo methods. In this approach, a sample from the posterior distribution is obtained by constructing a Markov chain with the target distribution as its stationary state, upon which parameters of the model are estimated and inferred. Presently, popular MCMC algorithms include the Metropolis-Hastings (MHs) algorithm, Gibbs sampling, and slice sampling, among others. In practice, the selection of an appropriate MCMC algorithm often depends on the nature of the target distribution and the dimensionality of the probability space. For instance, the MH algorithm is typically well-suited for low-dimensional probability distributions, whereas Gibbs sampling is favored for high-dimensional scenarios. In this study, we couple the MH algorithm with Gibbs sampling to harness the synergistic benefits of these two methodologies, thereby reducing sampling complexity.

According to Equation (29), the posterior distributions of α and β are as follows:


$$\pi (\alpha |x^{*},\beta )\propto \alpha^{\eta_{\text{1}}+n-1}e^{-\psi_{\text{1}}\alpha }\prod_{i=1}^{n}e^{\alpha (1-e^{x_{i}^{-\beta }})}$$ (33)

$$\pi (\beta |x^{*},\beta )\propto \beta^{\eta_{\text{2}}+n-1}e^{-\psi_{\text{2}}\beta }\prod_{i=1}^{n}x_{i}^{-\beta -1}e^{\alpha (1-e^{x_{i}^{-\beta }})}e^{x_{i}^{-\beta }}.$$ (34)

Equations (33) and (34) demonstrate that the posterior distribution of α and β is implicit, necessitating the application of mixed Gibbs sampling for the extraction of samples from this distribution. The iterative steps for implementing the mixed Gibbs sampling algorithm are delineated as follows:

(1) : Set the initial values of the parameters $\alpha =\alpha^{\left(0\right)}$, $\beta =\beta^{\left(0\right)}$.
(2) : Sample $\alpha^{\left(i+1\right)}$ from the distribution $\pi (\alpha |x^{*},\beta )$ using the MH algorithm. Choose the normal distribution $N(\alpha_{j},\mu_{\alpha }^{2})$ as the proposal distribution for α, where $\alpha_{j}$ represents the current state of α and $\mu_{\alpha }^{2}$ represents its variance.
  (i) A sample $\alpha^{\prime }$ is selected from the $N(\alpha_{j},\mu_{\alpha }^{2})$ and resampled when $\alpha^{\prime }\leq 0$. The acceptance probability is then calculated based on $\alpha^{\prime }$:
    $$\begin{array}{c} p(\alpha_{j},\alpha^{\prime })=\min\left\{1,\frac{\pi (\alpha^{\prime }|x^{*},\beta^{\left(i\right)})}{\pi (\alpha_{j}|x^{*},\beta^{\left(i\right)})}\right\}\\ =\min\left\{1,\frac{{\left(\alpha^{\prime }\right)}^{\eta_{1}+n-1}e^{-\psi_{1}\alpha^{\prime }}\prod_{i=1}^{n}e^{\alpha^{\prime }(1-e^{x_{i}^{-\beta^{\left(i\right)}}})}}{{\alpha_{j}}^{\eta_{1}+n-1}e^{-\psi_{1}\alpha_{j}}\prod_{i=1}^{n}e^{\alpha_{j}(1-e^{x_{i}^{-\beta^{\left(i\right)}}})}}\right\}. \end{array}$$
  (ii) Sample $\tau_{1}$ is taken from the uniform distribution U (0,1), so that:
    $$\alpha_{j+1}=\{\begin{array}{c} {}*20c\alpha^{\prime },\tau_{1}\leq p(\alpha_{j},\alpha^{\prime })\\ \alpha_{j},\tau_{1}>p(\alpha_{j},\alpha^{\prime }) \end{array}$$
  (iii) Let *j = j + 1*, return (i) to continue the above step.
  (3): Sample $\beta^{\left(i+1\right)}$ from the distribution $\pi (\beta |x^{*},\alpha )$ using the MH algorithm. Choose the normal distribution $N(\beta_{j},\mu_{\beta }^{2})$ as the proposal distribution for β, where $\beta_{j}$ represents the current state of β and $\mu_{\beta }^{2}$ represents its variance.

(i) A sample $\beta^{\prime }$ is selected from the $N(\beta_{j},\mu_{\beta }^{2})$ and resampled when $\beta^{\prime }\leq 0$. The acceptance probability is then calculated based on $\beta^{\prime }$:
  $$\begin{array}{c} p(\beta_{j},\beta^{\prime })=\min\left\{1,\frac{\pi (\beta^{\prime }|x^{*},\alpha^{\left(i+1\right)})}{\pi (\beta_{j}|x^{*},\alpha^{\left(i+1\right)})}\right\}\\ =\min\left\{1,\frac{{\left(\beta^{\prime }\right)}^{\eta_{2}+n-1}e^{-\psi_{2}\beta^{\prime }}\prod_{i=1}^{n}x_{i}^{-\beta^{\prime }-1}e^{\alpha^{\left(i+1\right)}(1-e^{x_{i}^{-\beta^{\prime }}})}e^{x_{i}^{-\beta^{\prime }}}}{{\beta_{j}}^{\eta_{2}+n-1}e^{-\psi_{2}\beta_{j}}\prod_{i=1}^{n}x_{i}^{-\beta_{j}-1}e^{\alpha^{\left(i+1\right)}(1-e^{x_{i}^{-\beta_{j}}})}e^{x_{i}^{-\beta_{j}}}}\right\}. \end{array}$$
(ii) Sample $\tau_{2}$ is taken from the uniform distribution U (0,1), so that:
  $$\beta_{j+1}=\{\begin{array}{c} {}*20c\beta^{\prime },\tau_{2}\leq p(\beta_{j},\beta^{\prime })\\ \beta_{j},\tau_{2}>p(\beta_{j},\beta^{\prime }) \end{array}.$$
(iii) Let *j = j + 1,* return (i) to continue the above step.

Samples $\alpha^{\left(i+1\right)}$ and $\beta^{\left(i+1\right)}$ of α and β are obtained through the above sampling steps, so the parameters of the TIWD model under PLF are Bayes estimator as:


$$\begin{array}{c} \hat{\varpi }(\alpha ,\beta )={\left[\frac{\sum {\mathrm{\backslash nolimits}}_{i=M_{0}+1}^{M}\varpi^{2}(\alpha^{\left(i\right)},\beta^{\left(i\right)})}{M-M_{0}}\right]}^{\frac{\text{1}}{\text{2}}}. \end{array}$$ (35)

In the above situation, we consider sampling under the condition of informative prior. When considering the non-informative prior, we only need to change the posterior distribution of α and β under the condition of informative prior to the posterior distribution under the condition of non-informative prior. It should be noted that during the iteration process of the Markov chain before reaching the target distribution, the sampling samples may be affected by the initial values. In order to ensure that the sampling samples used in the analysis can reliably represent the probability distribution being studied, we need to discard some samples and use $M_{0}$ to represent them.

### 5.3. AD estimation

AD estimation is a statistical test usually used to determine whether a set of data conforms to a particular probability distribution model, based on the difference between the cumulative and empirical distribution functions of the data. This test method was proposed by Anderson and Darling [29] in 1952. Compared with the traditional goodness of fit test, AD test has some unique advantages. It analyzes the cumulative function of the distribution, thus avoiding the direct analysis of the PDF. This makes the AD test more flexible in practical applications, adapting to a wide variety of data types and distributions. In the existing literature, many researchers used the AD estimation method to test the goodness of fit, so as to understand and analyze the data. For example, Alsadat et al [30] used AD estimation to estimate the reliability of unit Gompertz distribution. Shafiq et al. [31] used AD test to measure the parametric efficiency of the semilog unit Gamboz type I distribution. Aboraya et al. [32] used AD estimation to estimate small samples in the new compound Lomax model. In this paper, the AD estimates for ${\hat{\alpha }}_{AD}$ and ${\hat{\beta }}_{AD}$ can be obtained by minimizing the α and β functions: In this paper, the AD estimates for ${\hat{\alpha }}_{AD}$ and ${\hat{\beta }}_{AD}$ can be obtained by minimizing the α and β functions:


$$A(\Theta )=-n-\frac{\text{1}}{n}\sum {\mathrm{\backslash nolimits}}_{i=1}^{n}\left(2i-1)[\log F(x_{i};\Theta )+\log\{1-F(x_{n+1-i};\Theta )\}\right]$$ (36)

Among them, Θ represents the parameters α and β.

In addition, we can also obtain ${\hat{\alpha }}_{AD}$ and ${\hat{\beta }}_{AD}$ by solving the following nonlinear equations:


$$\begin{array}{c} \frac{\partial A(\Theta )}{\partial \alpha }=-\frac{\text{1}}{n}\sum {\mathrm{\backslash nolimits}}_{i=1}^{n}(2i-1)\left[\frac{\vartheta_{1}(x_{i};\Theta )}{F(x_{i};\Theta )}-\frac{\vartheta_{1}(x_{n+1-i};\Theta )}{1-F(x_{n+1-i};\Theta )}\right]=0 \end{array}$$ (37)

$$\begin{array}{c} \frac{\partial A(\Theta )}{\partial \beta }=-\frac{\text{1}}{n}\sum {\mathrm{\backslash nolimits}}_{i=1}^{n}(2i-1)\left[\frac{\vartheta_{2}(x_{i};\Theta )}{F(x_{i};\Theta )}-\frac{\vartheta_{2}(x_{n+1-i};\Theta )}{1-F(x_{n+1-i};\Theta )}\right]=0 \end{array}$$ (38)

Here


$$\vartheta_{1}(x_{i};\Theta )=e^{\alpha (1-e^{{x_{i}}^{-\beta }})}(1-e^{{x_{i}}^{-\beta }})$$ (39)

$$\vartheta_{2}(x_{i};\Theta )=\alpha x_{i}^{-\beta }e^{x_{i}^{-\beta }}e^{\alpha (1-e^{x_{i}^{-\beta }})}\ln x_{i}$$ (40)

### 5.4. CVM estimation

The CVM estimation is an important alternative to the Kolmogorov-Smirnov test, proposed by Harald Cramer and Richard von Mises, and it holds a significant place in statistics. This test offers a flexible and effective statistical method to verify whether a sample set conforms to a specific distribution by quantifying the discrepancy between the CDF of the sample and the CDF of the hypothetical distribution, thus providing an indepth comparison and evaluation of the hypothesis that the sample data set follows a particular distribution. As the CVM test is a non-parametric test, it possesses wide applicability and flexibility for small sample sizes, rendering it extensively used across various fields. In current literature, the CVM test is widely applied to validate various statistical models and data distributions. For instance, Wang and Zhu [33] employed CVM estimation to test the fitness of the lognormal distribution for power functions, corroborating the method with real-world data. Kutzker et al. [34] utilized the CVM test to assess the induction class of conditional distribution functions, while Cavaliere et al. [35] utilized the CVM test statistic to verify the correct specification of conditional variance functions within the GARCH model. Anis et al. [36] considered the application of the CVM test for parameter estimation in the Rayleigh distribution. These studies underscore the practical significance and broad applicability of the CVM test across numerous fields of research. In this paper, the CVM estimates for ${\hat{\alpha }}_{CVM}$ and ${\hat{\beta }}_{CVM}$ can be obtained by minimizing the α and β functions:


$$\begin{array}{c} C(\Theta )=\frac{\text{1}}{12n}+\sum {\mathrm{\backslash nolimits}}_{i=1}^{n}{\left[F(x_{i};\Theta )-\frac{2i-1}{2n}\right]}^{2} \end{array}$$ (41)

In addition, we can also obtain ${\hat{\alpha }}_{CVM}$ and ${\hat{\beta }}_{CVM}$ by solving the following nonlinear equations:


$$\begin{array}{c} \frac{\partial C(\Theta )}{\partial \alpha }=2\sum {\mathrm{\backslash nolimits}}_{i=1}^{n}\left[F(x_{i};\Theta )-\frac{2i-1}{2n}\right]\vartheta_{1}(x_{i};\Theta )=0 \end{array}$$ (42)

$$\begin{array}{c} \frac{\partial C(\Theta )}{\partial \beta }=2\sum {\mathrm{\backslash nolimits}}_{i=1}^{n}\left[F(x_{i};\Theta )-\frac{2i-1}{2n}\right]\vartheta_{2}(x_{i};\Theta )=0 \end{array}$$ (43)

Among them, $\vartheta_{j}(x_{i};\Theta )$, j = 1, 2 can be obtained from Equations (39) and (40).

### 5.5. OLS estimation

OLS is an estimation method used to estimate unknown parameters in linear regression models. It does this by minimizing the sum of squares of the residuals observed between the dependent and predictor variables in a given data set. Owing to its simple principle, strong optimality, and high efficiency, the OLS method has become an important tool for data analysis and is widely used in economics, finance, engineering, and other fields [37–40]. In this paper, the OLS estimates for ${\hat{\alpha }}_{OLS}$ and ${\hat{\beta }}_{OLS}$ can be obtained by minimizing the α and β functions:


$$\begin{array}{c} L(\Theta )=\sum {\mathrm{\backslash nolimits}}_{i=1}^{n}{\left[F(x_{i};\Theta )-\frac{i}{n+i}\right]}^{2} \end{array}$$ (44)

In addition, we can also obtain ${\hat{\alpha }}_{OLS}$ and ${\hat{\beta }}_{OLS}$ by solving the following nonlinear equations:


$$\begin{array}{c} \frac{\partial L(\Theta )}{\partial \alpha }=2\sum {\mathrm{\backslash nolimits}}_{i=1}^{n}\left[F(x_{i};\Theta )-\frac{i}{n+i}\right]\vartheta_{1}(x_{i};\Theta )=0 \end{array}$$ (45)

$$\begin{array}{c} \frac{\partial L(\Theta )}{\partial \beta }=2\sum {\mathrm{\backslash nolimits}}_{i=1}^{n}\left[F(x_{i};\Theta )-\frac{i}{n+i}\right]\vartheta_{2}(x_{i};\Theta )=0 \end{array}$$ (46)

Among them, $\vartheta_{j}(x_{i};\Theta )$, j = 1, 2 can be obtained from Equations (39) and (40).

Due to the complex forms of Equations (37), (38), (42), (43), (45), and (46), it is difficult to solve them directly. Therefore, we use numerical methods to solve these equations. As an efficient numerical analysis method, Newton Raphson method has significant advantages in solving root problems of nonlinear equations. Therefore, in this paper, we use Newton Raphson method to numerically solve these complex equations in order to obtain accurate solutions.

## 6. Simulation

To evaluate the uncertainty and complexity of the TIWD model, enhance the accuracy of statistical inference, and improve the efficiency of data processing, Matlab software is employed to compute various entropy measures under the TIWD model. We set the parameters α=1,1.5,2,2.5,3, β=0.025,0.050,0.075,0.1 and entropy parameters λ=0.9,1.1, with their specific values presented in Tables 1 and 2, respectively.

**Table 1:** Entropy measurement of TIWD model with different parameters when λ=0.9.

α	β	$H_{S}$	$H_{R}$	$H_{T}$	$H_{M}$
1.00	**0.025**	21.1757	47.1425	1105.2462	5.7138
**1.50**		25.4184	49.5477	1408.4972	8.3640
**2.00**		26.9674	50.9261	1618.1466	9.2164
**2.50**		27.1552	51.7492	1757.8187	9.5706
**3.00**		26.5927	52.2254	1844.0466	9.7425
**1.00**	**0.050**	19.4655	40.9184	588.4983	6.4764
**1.50**		24.6344	44.1064	813.2202	7.9457
**2.00**		28.1475	46.2812	1013.2135	8.6357
**2.50**		30.7051	47.9050	1193.6202	9.0214
**3.00**		32.6392	49.1830	1357.6940	9.2610
**1.00**	**0.075**	15.4174	32.9987	261.0916	6.1260
**1.50**		19.5671	36.2463	365.1102	7.3510
**2.00**		22.5914	38.5190	460.8253	8.0116
**2.50**		24.9623	40.2591	550.3123	8.4232
**3.00**		26.9036	41.6635	634.7943	8.7030
**1.00**	**0.100**	12.5055	25.6332	119.7886	5.7262
**1.50**		15.7605	28.7103	166.5519	6.8182
**2.00**		18.1643	30.8865	209.4740	7.4513
**2.50**		20.0741	32.5672	249.6423	7.8687
**3.00**		21.6594	33.9341	287.6741	8.1660

**Table 2:** Entropy measurement of TIWD model with different parameters when λ=1.1.

α	β	$H_{S}$	$H_{R}$	$H_{T}$	$H_{M}$
1.00	**0.025**	21.1757	8.4718	5.7138	1105.2462
**1.50**		25.4184	18.1033	8.3640	1408.4972
**2.00**		26.9674	25.4640	9.2164	1618.1466
**2.50**		27.1552	31.4801	9.5706	1757.8187
**3.00**		26.5927	36.5950	9.7425	1844.0466
**1.00**	**0.050**	19.4655	10.4311	6.4764	588.4983
**1.50**		24.6344	15.8266	7.9457	813.2202
**2.00**		28.1475	19.9197	8.6357	1013.2135
**2.50**		30.7051	23.2426	9.0214	1193.6202
**3.00**		32.6392	26.0506	9.2610	1357.6940
**1.00**	**0.075**	15.4174	9.4829	6.1260	261.0916
**1.50**		19.5671	13.2839	7.3510	365.1102
**2.00**		22.5914	16.1528	8.0116	460.8253
**2.50**		24.9623	18.4721	8.4232	550.3123
**3.00**		26.9036	20.4252	8.7030	634.7943
**1.00**	**0.100**	12.5055	8.5009	5.7262	119.7886
**1.50**		15.7605	11.4514	6.8182	166.5519
**2.00**		18.1643	13.6700	7.4513	209.4740
**2.50**		20.0741	15.4583	7.8687	249.6423
**3.00**		21.6594	16.9607	8.1660	287.6741

Then, this paper deeply analyzes the ML estimation, Bayesian estimation and other three estimation methods in TIWD model parameter estimation through simulation. Specifically, we first set the initial values of different parameters, α=0.5(1,2),β=1 and α=0.8,β=1(2,3) respectively, and selected the sample size n = 20, 30, 50, 80, 100 for 1000 cycles of simulation. In order to obtain ML estimation and other three estimation methods, the parameter average estimates (AEs) and the corresponding MSEs are obtained. In order to further evaluate the accuracy of each parameter estimation method, we also calculate the CVs of the parameters under each method. The CV is an important statistical indicator used to measure the relative dispersion of data, which eliminates the influence of different units or means on the dispersion by calculating the ratio of standard deviation to mean. Detailed results are shown in Tables 3 and 4. In the discussion of Bayesian estimation, since its performance is affected by the selection of prior distributions, we discuss it as a separate part. In this part, we consider two cases of informative prior and non-informative prior, and set the initial parameter values as α=1(1.5,2),β=1 and α=0.8,β=1(2,3), and keep the sample size unchanged. Through 1000 cycle simulation, we adopted the hyperparameter $\eta_{1}=\psi_{1}=\eta_{2}=\psi_{2}=1$ under the informative prior, and applied MCMC algorithm to carry out 5000 iterations, among which the first 500 iterations were used as the combustion period to eliminate the influence of the initial value, thus obtaining the AEs and MSEs of parameter Bayesian estimation, refer to Tables 5 and 6. It should be noted that for the sake of simplicity, we refer to INF and Non-INF as Bayesian estimates under informative prior and non-informative prior, respectively. To further analyze the performance of the estimation methods used, we set θ=0.05 and θ=0.1, construct ACIs for ML estimates at different confidence levels of 100(1−θ, and calculate the coverage probability (CP) and average width (AW) of the parametric confidence intervals. The specific results are shown in Table 7.

**Table 3:** When β=1, α takes the AEs of ML estimation, AD, CVM, OLS and the corresponding MSEs and CVs at different initial values.

α	n		ML estimation		AD			CVM				OLS		
			AE	MSE	CV	AE	MSE	CV	AE	MSE	CV	AE	MSE	CV
0.5	20	α	0.5094	0.0199	0.2765	0.5239	0.0155	0.2329	0.5262	0.0227	0.2821	0.4557	0.2066	0.9927
		β	1.0727	0.0407	0.1754	1.0190	0.0266	0.1589	1.0830	0.0661	0.2247	0.9317	0.2484	0.5299
	30	α	0.5041	0.0118	0.2153	0.4863	0.0096	0.1992	0.4958	0.0163	0.2577	0.5828	0.1410	0.6283
		β	1.0514	0.0245	0.1406	1.0259	0.0166	0.1231	1.0064	0.0446	0.2097	1.3252	0.2242	0.2597
	**50**	α	0.5070	0.0065	0.1580	0.4920	0.0071	0.1700	0.5119	0.0096	0.1904	0.5218	0.0711	0.5093
		β	1.0296	0.0140	0.1114	1.0067	0.0139	1.0067	1.0031	0.0216	0.1466	1.0929	0.0885	0.2586
	**80**	α	0.5015	0.0041	0.1276	0.5035	0.0041	0.1275	0.4837	0.0048	0.1390	0.3668	0.0447	0.4475
		β	1.0209	0.0067	0.0777	1.0057	0.0100	0.0993	1.0367	0.0160	0.1166	1.2450	0.0845	0.1257
	**100**	α	0.5001	0.0032	0.1124	0.5183	0.0037	0.1124	0.5028	0.0028	0.1058	0.4437	0.0136	0.2307
		β	1.0126	0.0054	0.0713	0.9945	0.0065	0.0807	1.0127	0.0088	0.0915	1.1700	0.0370	0.0769
**1**	**20**	α	1.0903	0.1063	0.2874	1.0207	0.0501	0.2184	1.1524	0.0868	0.2188	1.2366	0.0917	0.1529
		β	1.1046	0.0658	0.2121	1.0085	0.0369	0.1902	1.1252	0.0895	0.2415	1.2122	0.1074	0.2060
	**30**	α	1.0512	0.0534	0.2144	1.0021	0.0236	0.1532	1.1283	0.0727	0.2101	1.1490	0.0493	0.1433
		β	1.0652	0.0332	0.1596	0.9835	0.0298	0.1746	1.1119	0.0606	0.1971	1.1460	0.0508	0.1498
	**50**	α	1.0257	0.0238	0.1482	0.9959	0.0179	0.1343	1.0412	0.0311	0.1647	1.1842	0.0402	0.0667
		β	1.0295	0.0165	0.1214	1.0043	0.0159	0.1255	1.0456	0.0253	0.1457	1.1760	0.0448	0.1000
	**80**	α	1.0126	0.0132	0.1128	0.9949	0.0118	0.1093	1.0269	0.0181	0.1285	1.1920	0.0400	0.0470
		β	1.0147	0.0094	0.0944	0.9999	0.0126	0.1121	1.0171	0.0170	0.1271	1.1900	0.0410	0.0588
	**100**	α	1.0156	0.0113	0.1037	1.0088	0.0092	0.0946	1.0101	0.0168	0.1279	1.0940	0.0106	0.0384
		β	1.0179	0.0076	0.0838	1.0014	0.0098	0.0991	1.0034	0.0103	0.1009	1.0920	0.0116	0.0513
**2**	**20**	α	2.3453	0.8089	0.3541	2.0257	0.1468	0.1887	2.2199	0.1771	0.1617	2.0417	0.2486	0.2433
		β	1.0914	0.0669	0.2217	1.0037	0.0636	0.2512	1.0877	0.0953	0.2722	1.0406	0.2495	0.4784
	**30**	α	2.2359	0.4314	0.2741	2.0193	0.1369	0.1830	2.1961	0.1521	0.1535	2.0340	0.1166	0.1670
		β	1.0701	0.0369	0.1672	1.0146	0.0426	0.2030	1.0691	0.0518	0.2029	1.0960	0.1600	0.3543
	**50**	α	2.1093	0.1743	0.1910	2.0240	0.1017	0.1571	2.1189	0.1151	0.1499	2.0100	0.0590	0.1207
		β	1.0338	0.0196	0.1314	1.0016	0.0271	0.1643	1.0378	0.0442	0.1993	1.0720	0.0900	0.2717
	**80**	α	2.0736	0.0951	0.1444	2.0236	0.0617	0.1222	2.0993	0.0854	0.1309	1.9470	0.0253	0.0770
		β	1.0188	0.0117	0.1045	1.0190	0.0132	0.1114	1.0286	0.0287	0.1625	1.0400	0.0400	0.1884
	**100**	α	2.0672	0.0730	0.1266	2.0414	0.0577	0.1159	2.0490	0.0787	0.1348	1.9590	0.0041	0.0251
		β	1.0210	0.0096	0.0938	1.0220	0.0108	0.0993	0.9893	0.0291	0.1722	1.0180	0.0100	0.0966

**Table 4:** When α=0.8, β takes the AEs of ML estimation, AD, CVM, OLS and the corresponding MSEs and CVs at different initial values.

β	n		ML estimation			AD			CVM			OLS		
			AE	MSE	CV	AE	MSE	CV	AE	MSE	CV	AE	MSE	CV
1	20	α	0.8506	0.0491	0.2537	0.7915	0.0347	0.2352	0.8750	0.0489	0.2378	1.2419	0.2402	0.1708
		β	1.0951	0.0580	0.2021	1.0345	0.0396	0.1894	1.0982	0.0706	0.2249	1.4700	0.2500	0.1160
	30	α	0.8229	0.0277	0.2003	0.8179	0.0250	0.1921	0.8491	0.0340	0.2094	1.2646	0.2348	0.1088
		β	1.0588	0.0288	0.1505	0.9896	0.0264	0.1639	1.0347	0.0356	0.1793	1.4730	0.2473	0.1042
	50	α	0.8180	0.0146	0.1460	0.8118	0.0126	0.1376	0.8273	0.0160	0.1494	1.0911	0.0891	0.0605
		β	1.0281	0.0155	0.1179	1.0003	0.0143	0.1197	1.0104	0.0186	0.1345	1.3790	0.1579	0.0866
	80	α	0.8113	0.0091	0.1165	0.8139	0.0076	0.1059	0.8267	0.0104	0.1194	0.8970	0.0103	0.0333
		β	1.0188	0.0088	0.0904	1.0046	0.0096	0.0973	1.0083	0.0119	0.1081	1.0940	0.0106	0.0384
	100	α	0.8025	0.0066	0.1016	0.7972	0.0065	0.1011	0.8125	0.0077	0.1066	0.8960	0.0100	0.0313
		β	1.0147	0.0067	0.0794	0.9991	0.0084	0.0918	1.0041	0.0116	0.1073	1.0960	0.0100	0.0255
1.5	20	α	0.8550	0.0505	0.2547	0.8152	0.0358	0.2313	0.8261	0.0434	0.2501	1.2037	0.2295	0.2143
		β	1.6415	0.1287	0.2008	1.5099	0.0781	0.1850	1.5638	0.1063	0.2044	1.9600	0.2500	0.1000
	30	α	0.8296	0.0282	0.1991	0.7911	0.0224	0.1887	0.8571	0.0320	0.1979	1.1675	0.1541	0.1182
		β	1.5899	0.0676	0.1535	1.5445	0.0544	0.1483	1.5770	0.0793	0.1718	1.8840	0.1600	0.0594
	50	α	0.8129	0.0155	0.1526	0.8253	0.0157	0.1487	0.8113	0.0168	0.1589	1.0905	0.0886	0.0591
		β	1.5550	0.0363	0.1173	1.4987	0.0287	0.1131	1.5371	0.0337	0.1170	1.7940	0.0900	0.0333
	80	α	0.8135	0.0089	0.1145	0.8027	0.0092	0.1196	0.8226	0.0091	0.1128	0.9934	0.0395	0.0465
		β	1.5198	0.0194	0.0908	1.4960	0.0167	0.0864	1.5603	0.0342	0.1120	1.6920	0.0400	0.0331
	100	α	0.8043	0.0071	0.1048	0.7899	0.0065	0.1016	0.8211	0.0082	0.1072	0.8980	0.0100	0.0222
		β	1.5223	0.0165	0.0830	1.5127	0.0154	0.0817	1.5545	0.0345	0.1142	1.5980	0.0100	0.0125
3	20	α	0.8540	0.0515	0.2580	0.8499	0.0401	0.2282	0.8674	0.0589	0.2688	1.1695	0.2213	0.2490
		β	3.3125	0.5475	0.2025	3.0189	0.1667	0.1351	3.0765	0.1662	0.1302	3.4800	0.2500	0.0402
	30	α	0.8349	0.0290	0.1997	0.8008	0.0238	0.1927	0.8493	0.0301	0.1958	1.1392	0.1562	0.1782
		β	3.1658	0.2570	0.1513	3.0088	0.1242	0.1171	3.0266	0.1547	0.1297	3.3920	0.1600	0.0235
	50	α	0.8134	0.0167	0.1580	0.7873	0.0139	0.1487	0.8108	0.0169	0.1597	1.0793	0.0891	0.0976
		β	3.1129	0.1501	0.1190	3.0069	0.1050	0.1077	3.0362	0.1121	0.1096	3.2940	0.0900	0.0181
	80	α	0.8121	0.0098	0.1213	0.8123	0.0091	0.1164	0.8170	0.0112	0.1280	0.9940	0.0394	0.0423
		β	3.0419	0.0823	0.0933	2.9836	0.0808	0.0951	3.0705	0.1021	0.1015	3.0940	0.0106	0.0136
	100	α	0.8089	0.0072	0.1042	0.7998	0.0068	0.1031	0.8155	0.0107	0.1257	0.8980	0.0100	0.0222
		β	3.0405	0.0620	0.0808	3.0173	0.0660	0.0849	2.9795	0.0802	0.0948	3.0980	0.0100	0.0064

**Table 5:** When β=1, the AEs and corresponding MSEs of Bayesian estimation when α takes different initial values.

α	n		INF		Non-INF	
			AE	MSE	AE	MSE
**1**	**20**	α	1.0899	0.0830	1.1028	0.1078
		β	1.0809	0.0491	1.0889	0.0577
	**30**	α	1.0647	0.0549	1.0702	0.0650
		β	1.0568	0.0310	1.0615	0.0341
	**50**	α	1.0481	0.0277	1.0487	0.0294
		β	1.0354	0.0181	1.0368	0.0191
	**80**	α	1.0245	0.0148	1.0246	0.0154
		β	1.0204	0.0103	1.0207	0.0105
	**100**	α	1.0187	0.0119	1.0186	0.0120
		β	1.0202	0.0087	1.0198	0.0088
**1.5**	**20**	α	1.6262	0.1963	1.7205	0.3739
		β	1.0726	0.0489	1.0952	0.0635
	**30**	α	1.6113	0.1370	1.6632	0.2017
		β	1.0631	0.0346	1.0745	0.0407
	**50**	α	1.5494	0.0621	1.5724	0.0745
		β	1.0406	0.0194	1.0457	0.0209
	**80**	α	1.5371	0.0397	1.5511	0.0439
		β	1.0138	0.0097	1.0172	0.0102
	**100**	α	1.5238	0.0284	1.5349	0.0314
		β	1.0158	0.0082	1.0188	0.0086
**2**	**20**	α	2.1290	0.3364	2.4116	1.0431
		β	1.0568	0.0463	1.1067	0.0738
	**30**	α	2.0980	0.2268	2.2555	0.4461
		β	1.0431	0.0310	1.0729	0.0408
	**50**	α	2.0651	0.1382	2.1403	0.1963
		β	1.0294	0.0178	1.0446	0.0213
	**80**	α	2.0360	0.0800	2.0763	0.0974
		β	1.0150	0.0106	1.0232	0.0118
	**100**	α	2.0363	0.0630	2.0690	0.0777
		β	1.0139	0.0081	1.0203	0.0089

**Table 6:** When α=0.8, the AEs and corresponding MSEs of Bayesian estimation when β takes different initial values.

β	n		INF		Non-INF	
			AE	MSE	AE	MSE
**1**	**20**	α	0.8556	0.0429	0.8535	0.0502
		β	1.0824	0.0478	1.0895	0.0540
	**30**	α	0.8448	0.0294	0.8406	0.0309
		β	1.0548	0.0275	1.0594	0.0302
	**50**	α	0.8199	0.0144	0.8171	0.0148
		β	1.0327	0.0152	1.0333	0.0157
	**80**	α	0.8191	0.0095	0.8177	0.0095
		β	1.0178	0.0089	1.0182	0.0090
	**100**	α	0.8122	0.0073	0.8100	0.0073
		β	1.0175	0.0071	1.0180	0.0073
**2**	**20**	α	0.8754	0.0481	0.8718	0.0575
		β	2.0999	0.1700	2.1881	0.2424
	**30**	α	0.8514	0.0289	0.8456	0.0311
		β	2.0593	0.1028	2.1110	0.1295
	**50**	α	0.8270	0.0148	0.8237	0.0156
		β	2.0397	0.0611	2.0665	0.0677
	**80**	α	0.8223	0.0101	0.8199	0.0103
		β	2.0311	0.0343	2.0497	0.0372
	**100**	α	0.8156	0.0070	0.8130	0.0071
		β	2.0208	0.0288	2.0345	0.0302
**3**	**20**	α	0.8648	0.0447	0.8600	0.0562
		β	3.0521	0.3242	3.2915	0.5655
	**30**	α	0.8523	0.0273	0.8479	0.0320
		β	3.0353	0.1907	3.1889	0.2842
	**50**	α	0.8253	0.0147	0.8205	0.0157
		β	3.0252	0.1346	3.1030	0.1597
	**80**	α	0.8157	0.0087	0.8117	0.0091
		β	3.0195	0.0733	3.0712	0.0861
	**100**	α	0.8173	0.0081	0.8144	0.0084
		β	3.0013	0.0580	3.0396	0.0639

**Table 7:** When β=1, the CP and AW of parameter confidence intervals at different confidence levels of 100(1−θ for different initial values of α.

α	n		θ=0.05		θ=0.1	
			CP	AW	CP	AW
**0.5**	**20**	α	0.9860	0.7702	0.9720	0.6795
		β	0.9960	1.0567	0.9920	0.9289
	**30**	α	0.9780	0.6161	0.9720	0.5455
		β	0.9950	0.8223	0.9890	0.7339
	**50**	α	0.9930	0.4736	0.9830	0.4234
		β	0.9960	0.6215	0.9920	0.5565
	**80**	α	0.9930	0.3730	0.9920	0.3350
		β	0.9970	0.4848	0.9910	0.4331
	**100**	α	0.9940	0.3349	0.9870	0.2987
		β	0.9970	0.4319	0.9900	0.3875
**1**	**20**	α	0.9890	1.4980	0.9850	1.3223
		β	0.9990	1.2166	0.9920	1.0894
	**30**	α	0.9930	1.1667	0.9860	1.0342
		β	0.9970	0.9612	0.9930	0.8534
	**50**	α	0.9960	0.8723	0.9940	0.7849
		β	0.9990	0.7176	0.9890	0.6457
	**80**	α	0.9940	0.6794	0.9890	0.6066
		β	0.9980	0.5633	0.9910	0.4995
	**100**	α	0.9960	0.6054	0.9880	0.5398
		β	0.9990	0.4963	0.9920	0.4431
**2**	**20**	α	0.9930	4.0388	0.9890	3.6749
		β	0.9990	1.2922	0.9950	1.1589
	**30**	α	0.9970	3.0227	0.9960	2.6982
		β	0.9950	1.0275	0.9920	0.9073
	**50**	α	0.9930	2.1677	0.9930	1.9694
		β	0.9970	0.7703	0.9890	0.6943
	**80**	α	0.9940	1.6614	0.9890	1.4929
		β	0.9990	0.6018	0.9900	0.5406
	**100**	α	0.9970	1.4667	0.9960	1.3076
		β	0.9970	0.5347	0.9880	0.4785

According to the above tables, the following conclusions can be drawn:

(1) When β and λ are fixed, the measures of entropy show an upward trend with the increase of α. When α and λ are fixed, the entropy measures tend to decrease with the increase of β.
(2) With the increase of sample size, both MSEs and CVs of TIWD model parameter estimates showed a decreasing trend, indicating that the accuracy of estimation increased with the increase of sample size.
(3) Among various parameter estimation methods, ML estimation shows superior performance in overall accuracy compared with the other three estimation methods. At the same time, the OLS estimation is generally superior to other estimation methods in terms of precision.
(4) In Bayesian estimation, Bayesian estimation based on informative prior is superior to Bayesian estimation based on non-informative prior in accuracy.
(5) With the increase of confidence level, CP and AW of confidence interval both show an increasing trend.

## 7. Real data analysis

In this section, we evaluate the applicability of the TIWD model in practical applications by empirically analyzing two sets of real data. The first set of data represents the fatigue life of metal components (in cycles) [41]. The second set of data reflects the survival time of patients with head and neck cancer [42]. The specific details of these two sets of data are detailed in Table 8.

**Table 8:** Fatigue life data of metal components and survival time length data of head and neck cancer patients.

Date										
**Ⅰ**	125	127	135	137	185	187	190	190	195	200
	212	242	245	255	283	316	327	355	373	386
	456	482	552	580	700	736	745	750	804	852
	884	977	1040	1066	1093	1114	1125	1300	1536	1583
	2208	2266	2834	3280	4707	5046				
**Ⅱ**	12.20	23.56	23.74	25.87	31.98	37	41.35	47.38	55.46	58.36
	63.47	68.46	78.26	74.74	81.43	84	92	94	110	112
	119	127	130	133	140	146	155	159	173	179
	194	195	209	249	281	319	339	432	469	519
	633	725	817	1776						

Before conducting in-depth analysis on the two sets of real data we provided, it is necessary to verify whether the real data conforms to the characteristics of algebraic decay. Whether the real data satisfies algebraic decay directly affects the accuracy and reliability of the TIWD model in statistical inference, which is the key to our subsequent research. Through Equation (7), we provide the following analysis:

When x→∞, there is $x^{-\beta }\to 0$, and $e^{x^{-\beta }}$ is expanded by Taylor expansion to obtain:

$e^{x^{-\beta }}\approx 1+x^{-\beta }$.

**Fig 6:**
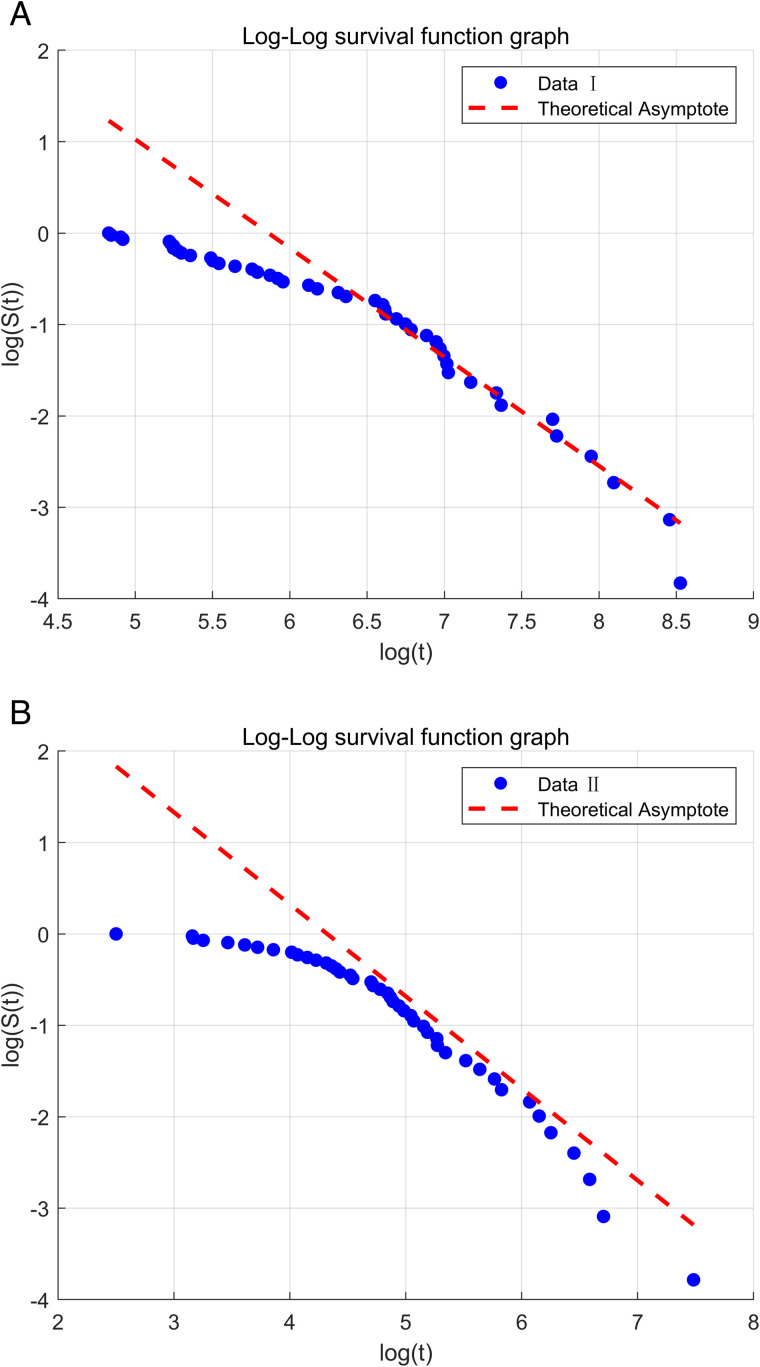
Log-Log SF plots under two sets of real data.

So there is $S(x)\approx 1-e^{-\alpha x^{-\beta }}\approx 1-(1-\alpha x^{-\beta })\approx \alpha x^{-\beta }$. That is to say, when x→∞, there is $S(x)\sim \alpha x^{-\beta }$. It indicates that S(x) exhibits algebraic decay characteristics. Next, we will further verify that the real data satisfies the algebraic decay characteristic by plotting the log-log SF graph. Please refer to Fig 6. From Fig 6, it can be observed that the tail data is closer to the theoretical asymptotic line. The blue data points gradually align with the red dotted line, indicating that the tail conforms to the asymptotic characteristic of algebraic decay, which is consistent with the thick-tailed characteristic of TIWD.

In order to further evaluate the performance of the TIWD model in fitting two sets of real data, we used Kolmogorov Smirnov (KS) test, Akaike information criterion (AIC), bias correction AIC (AICc) [43], and Bayesian information criterion (BIC) [44] to quantitatively evaluate the model. The calculation formulas for AIC, AICc, and BIC are as follows:47


AIC=−2ln(L)+2k,

$$AICc=AIC+\frac{2k(k+1)}{n-k-1},$$

BIC=−2ln(L)+kln(n).

**Fig 7:**
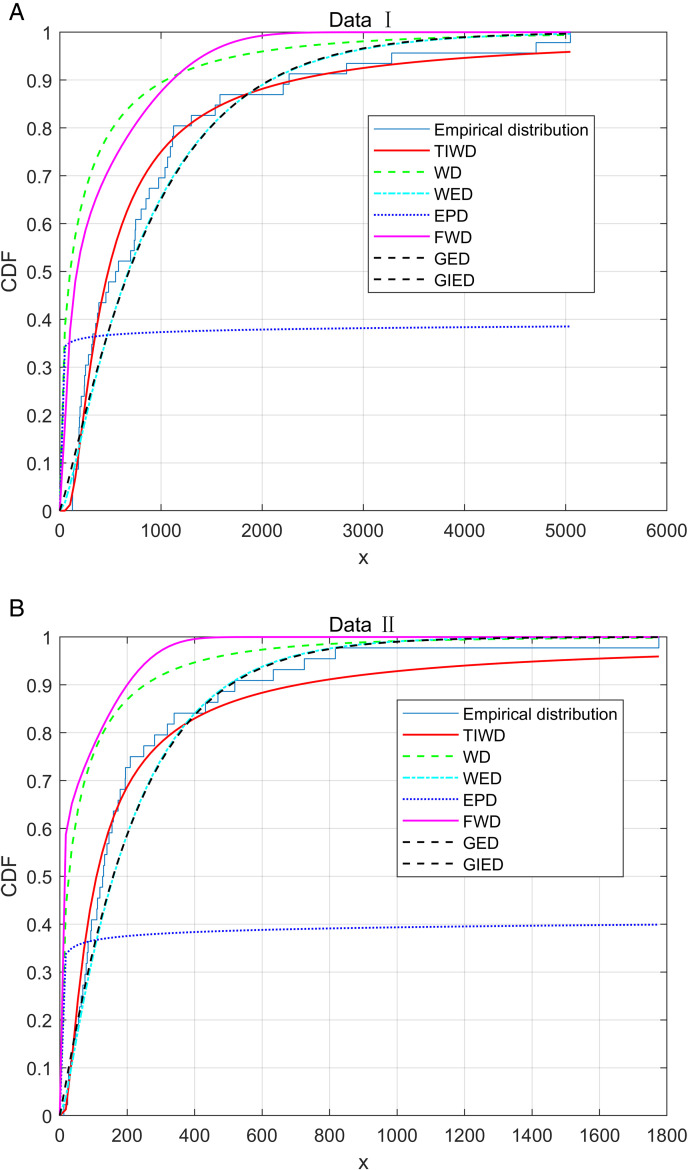
Empirical distribution plots based on real data and CDF plots of the TIWD model.

**Fig 8:**
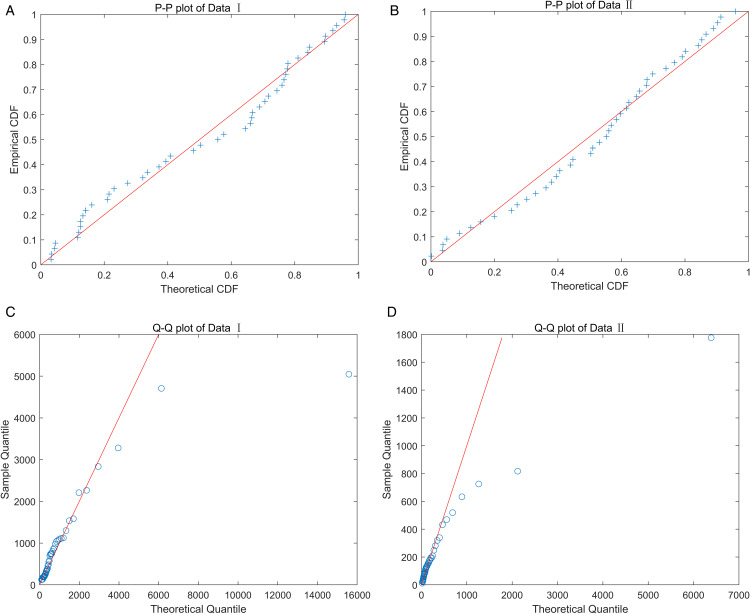
PP and QQ plots.

Here, ln(L) represents the log-likelihood function of the parameters given the data, *k* indicates the number of parameters in the model, and *n* represents the sample size. During this process, we use the *p*-value of the KS test as the key indicator to evaluate the goodness of fit of the model. To further highlight the outstanding performance of the TIWD model, we compared it with the WD, weighted exponential distribution (WED) [45], exponentiated Pareto distribution (EPD) [46], FWD [47], generalized exponential distribution (GED) [48], and generalized inverse exponential distribution (GIED) [49]. The specific test results are detailed in Table 9. To visually demonstrate the advantages of the TIWD model in fitting the two sets of data, we plotted the empirical distribution function of the real data and the CDFs of each model, as shown in Fig 7. Additionally, we created PP plots and QQ plots of the TIWD model for the two sets of real data to more clearly reveal the fitting effect. The relevant graphical displays are presented in Fig 8.

**Table 9:** ML estimates, AIC test values, AICc test values, BIC values, KS test values, and corresponding p-values of each model parameter under two sets of real data.

Date	Model	α	β	AIC	AICc	BIC	KS	p
**Ⅰ**	**TIWD**	1074.9606	1.1908	715.3629	715.6420	719.0202	0.1006	0.3944
	**WD**	207.8416	0.5155	777.2233	777.5024	780.8806	0.5150	2.5322E-11
	**WED**	11.8622	0.0011	721.8395	722.1186	725.4968	0.1320	0.2011
	**EPD**	0.0002	0.1486	1.0237E + 03	1.0240E + 03	1.0274E + 03	0.6149	1.7002E-12
	**FWD**	84.8485	0.0008	764.1916	764.4707	767.8489	0.4121	1.6389E-07
	**GED**	1.1752	0.0012	725.5515	725.8305	729.2087	0.1274	0.2247
	**GIED**	453.3287	0.2847	786.7947	787.0738	790.4520	0.5427	1.7002E-12
**Ⅱ**	**TIWD**	77.6807	1.0071	563.3477	563.6404	566.9161	0.0843	0.5351
	**WD**	52.0149	0.5280	610.0105	610.3032	613.5789	0.4400	3.9812E-08
	**WED**	15.7931	0.0047	565.4368	565.7295	569.0052	0.1491	0.0047
	**EPD**	0.0008	0.1794	808.1118	808.4045	811.6802	0.6009	2.0662E-19
	**FWD**	3.5635	0.0043	587.2750	587.5677	590.8434	0.5680	4.6713E-13
	**GED**	1.0724	0.0047	567.9122	568.2049	571.4806	0.1496	0.1395
	**GIED**	84.8538	0.1407	671.4744	671.7671	675.0428	0.6992	2.0662E-19

Through these specialized analysis charts, we can clearly observe that TIWD has a good fitting effect, which enables us to more accurately evaluate the performance of the TIWD model in practical applications. We used all the estimation methods in this study to obtain the parameter values and corresponding KS test values and *p*-values for these two sets of real data, as shown in Table 10. According to Table 10, the ML estimation performance is the best in real Data I, and the Bayesian estimation performance under informative prior is the best in real Data II.

**Table 10:** TIWD parameter values of various estimation methods under real data.

Data		ML estimation	AD	CVM	OLS	Bayesian estimation	
						INF	Non-INF
**Ⅰ**	α	1074.9606	1074.9679	1094.3885	1141.8558	1156.1219	1624.4739
	β	1.1908	4.9521	1.4006	1.0433	1.0003	1.2172
	**KS**	0.1005	0.9783	0.3727	0.3333	0.4459	0.1567
	**p**	0.3948	5.7970e-39	2.8122e-06	3.6394e-05	1.1378e-08	0.1044
**Ⅱ**	α	77.6807	75.7171	70.7293	88.7511	81.1761	81.9302
	β	1.0071	7.4586	1.2760	0.8656	0.9967	0.9832
	**KS**	0.0843	0.9773	0.4665	0.3331	0.0772	0.0995
	**p**	0.5354	3.1591e-37	4.8031e-09	5.7410e-05	0.5920	0.4188

In addition to the two sets of real data mentioned above, the durability test data of deep groove ball bearings under working conditions and the dataset of infection occurrence times of hemodialysis patients within several months can also be considered for analysis. These two sets of data have good fitting characteristics with the TIWD model, so similar research can be conducted on them to further expand the application scope of the TIWD model.

## 8. Conclusions

In this study, we conducted a series of transformations on the IWD, proposed a novel TIWD, and conducted in-depth analysis of the statistical and mathematical properties of this distribution model. Firstly, we discussed the mathematical properties and statistical characteristics of TIWD. Subsequently, we used five estimation methods to estimate the parameters of the model and conducted a comprehensive evaluation of these estimation methods through simulation. The research results indicate that Bayesian estimation performs outstandingly in parameter estimation accuracy with the introduction of prior information, effectively reducing the impact of prior on the results. To verify the practical application performance of the TIWD model, we selected two sets of real data for empirical testing. Through goodness of fit testing, we observed that the TIWD model performed better in terms of data fitting accuracy and adaptability compared to other distribution models. Previous studies have shown that traditional IWD models have certain limitations in processing complex data in certain situations, and cannot accurately describe the distribution characteristics of specific data. The TIWD model obtained through mathematical transformation introduces new parameters, improves the flexibility and adaptability of the model, and can effectively improve the fitting effect of the model on data. From a theoretical perspective, the TIWD model is a further extension and generalization of the IWD model. It not only enriches the probability distribution theory system and improves the theoretical tools for describing complex data distributions, but also provides a deeper understanding of the properties and characteristics of the IWD model and its extended models. At the same time, the proposal of the TIWD model has strengthened the connection between statistics and other disciplines, promoted the development of interdisciplinary research, and provided new methodological support for solving complex problems in practice.

Although the TIWD model has certain advantages in expanding the applicability and fitting accuracy of traditional IWD models, it still has certain limitations. Firstly, due to the introduction of new parameters in the TIWD model, parameter estimation is more complex compared to traditional IWD models. When analyzing ML estimation, there may be difficulties in convergence, often requiring consideration of more numerical methods for computation. Secondly, the TIWD model requires specific data for calibration and validation when fitting real data, otherwise it will affect the performance of the model. Therefore, collecting and organizing data that meets the requirements of the model may require a significant amount of time and manpower, and may face difficulties in obtaining data in practical applications. Finally, due to the fixed parameter structure of the TIWD model, it may exhibit poor adaptability to new data in some practical application scenarios, making it difficult to flexibly adapt to different conditions and requirements. Continuous adjustments are needed to adapt to new scenarios.

Given the limitations of the TIWD model, future research needs to develop more efficient numerical calculation methods to obtain parameter estimates. Before performing data fitting, it is necessary to preprocess the data to remove outliers and improve data quality. At the same time, clarify the data standards that meet the requirements of the TIWD model, providing guidance for collecting and organizing data to reduce time and manpower. Finally, we should actively explore the research of TIWD models in emerging fields such as artificial intelligence and big data analysis. By expanding the research field and discovering the advantages and disadvantages of TIWD in different fields, the model can be improved and perfected, providing new ideas and methods for data analysis in various fields.

## Supporting information

S1 File Support information explanation. (TIF)

## Abbreviations

WD: Weibull distribution
IWD: Inverse WD
FWD: Flexible WD
TIWD: Transformed IWD
PDF: Probability Density Function
CDF: Cumulative Distribution Function
SF: Survival Function
HF: Hazard Function
ML: Maximum Likelihood
AD: Anderson Darling
CVM: Cramer-von-Mises
OLS: Ordinary Least Squares
MSE: Mean Square Error
CV: Coefficient of Variation
ACI: Asymptotic Confidence Interval
PLF: Precautionary Loss Function
MH: Metropolis-Hasting
AE: Average Estimate
CP: Coverage Probability
AW: Average Width
WED: Weighted Exponential Distribution
EPD: Exponentiated Pareto Distribution
GED: Generalized Exponential Distribution
GIED: Generalized Inverse Exponential Distribution
