# Supplementary figures and images for: A novel extended inverse Weibull distribution: Statistical analysis and application
Source: PLoS One. 2025 Oct 28;20(10):e0335555. doi: 10.1371/journal.pone.0335555 (PMC12562005; doi:10.1371/journal.pone.0335555)

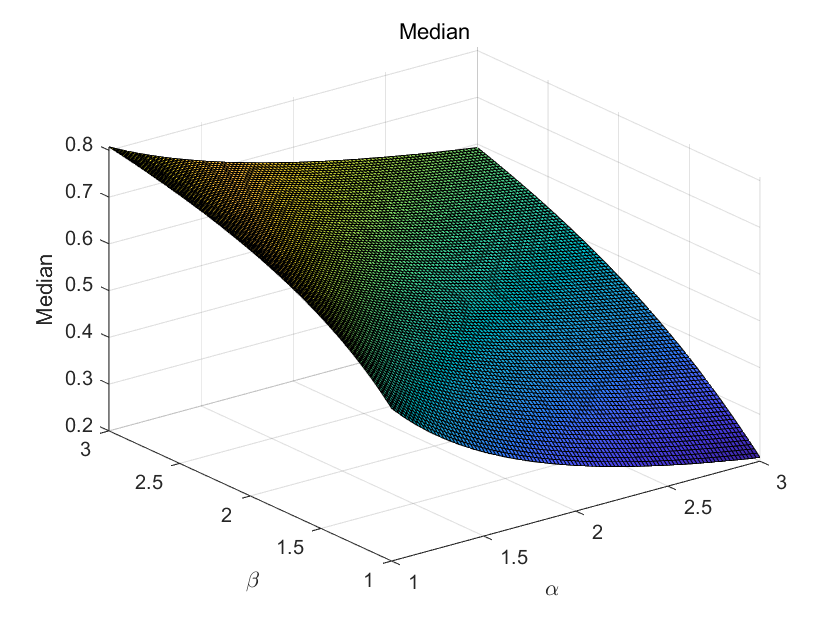

Supplement: S1 File — (TIF) [file pone.0335555.s001.tif]
